# Exosomes derived from M2 macrophages induce angiogenesis to promote wound healing

**DOI:** 10.3389/fmolb.2022.1008802

**Published:** 2022-10-11

**Authors:** Leifeng Lyu, Yuanqing Cai, Guangyang Zhang, Zhaopu Jing, Jialin Liang, Rupeng Zhang, Xiaoqian Dang, Chen Zhang

**Affiliations:** Department of Orthopedics, The Second Affiliated Hospital of Xi’an Jiaotong University, Xi’an, China

**Keywords:** exosomes, M2 macrophages, angiogenesis, wound healing, tissue repair

## Abstract

There is an urgent clinical need for an appropriate method to shorten skin healing time. Among most factors related to wound healing, M2 macrophages will be recruited to the wound area and play a pivotal role in a time-limiting factor, angiogenesis. The exploration of exosomes derived from M2 in angiogenesis promotion is an attractive research field. In this project, we found that exosomes from M2 (M2-EXO) promoted the angiogenic ability of HUVECs *in vitro*. With a series of characteristic experiments, we demonstrated that M2-EXO inhibited PTEN expression in HUVECs by transferring miR-21, and further activated AKT/mTOR pathway. Then, using a full-thickness cutaneous wound mice model, we demonstrated that M2-EXO could be used as a promotor of angiogenesis and regeneration *in vivo*. Furthermore, M2-EXO-treated skin wounds exhibited regeneration of functional microstructures. These results demonstrate that M2-EXO can be used as a promising nanomedicine strategy for therapeutic exploration of skin healing with the potential to be translated into clinical practice.

## Introduction

As the human body’s largest organ, our skin protects us from environmental stimuli (Gravitz, 2018). However, the skin is frequently damaged by accidental trauma, burns, or surgical procedures and develops wound infections or scarring because of delayed healing (Broughton et al., 2006). Therefore, shortening the healing time after skin damage and restoring its structural integrity is urgently needed in clinical practice (Chin et al., 2019). Except for medical needs, impaired wound healing has also been a social and economic problem. It is reported that the global wound care market has exceeded more than $20 billion (Nussbaum et al., 2018). Although various therapeutic attempts have been made to promote wound healing over the past decades, challenges still need to be addressed, involving scar formation, abnormal tissue hyperplasia, and ischemia of wound tissue. Most tissue injuries involve localized vascular system disruption, and the vascular system’s reconstruction is essential for tissue repair; this process of growing new blood vessels from existing vessels is called angiogenesis (Gurevich et al., 2018). Angiogenesis and blood supply restoration are considered time-limiting factors in wound healing (Morel et al., 2006). Therefore, therapies to improve angiogenesis in wounds are critical and feasible.

Angiogenesis is a critical mechanism in wound healing involving the dynamic coordination of extracellular matrix, cells, and cytokines (Greaves et al., 2015). Extensive studies have shown that macrophages are closely associated with angiogenesis at the wound site (Hesketh et al., 2017). These healing-associated macrophages are named alternatively activated or M2 macrophages (Mahdavian Delavary et al., 2011). In addition, during the later stages of skin repair, wound macrophages upregulate Metallo-matrix protease (MMP) to remodel the extracellular matrix to prevent scar formation (Okuno et al., 2011). Therefore, some research focused on regulating the number or activation level of M2 at the wound site to improve wound healing and got excellent results (Shook et al., 2016; Toita et al., 2021). However, few studies have applied M2-EXO to skin healing. Exosomes are considered cellular fingerprints, due to their close correlation with the derived mother cells, which determines that these natural vesicles can trigger specific biological processes at the target site (Hettich et al., 2020). Therefore, it is reasonable to assume that M2-EXO may induce the angiogenesis at the wound site. Exosomes, the pivotal regulator in the intercellular communication process (Colombo et al., 2014), have great potential in skin wound healing. For instance, exosomes derived from human umbilical cord blood stem cells, bone marrow mesenchymal stem cells, adipose mesenchymal stem cells, *etc.*, have been proved owe excellent repair effects (Shabbir et al., 2015; Hu et al., 2018; Zhang et al., 2018; An et al., 2021). In conclusion, exploring the role of M2-EXO in promoting angiogenesis and the exact mechanism under the promotion effect is of great interest.

In this project, we found that M2-EXO can promote the angiogenic ability of HUVEC *in vitro*. We also demonstrated that miR-21-5p was transferred to HUVEC *via* exosomes from M2 and worked as a critical mediator within M2-EXO, inhibiting PTEN and activating AKT/mTOR signaling followed by regulating endothelial cell function. Besides, we constructed a full thickness cutaneous wound mice model to evaluate the influence of M2-EXO treatment on wound healing. Results showed that the injection of M2-EXO into murine real skin wounds enhanced angiogenesis and accelerated skin healing. Overall, Our study provides a new perspective on the promotion of angiogenesis by M2 and a promising cell-free therapeutic strategy for wound healing.

## Mateials and methods

### Cell culture

HUVEC was purchased from Shanghai Cell Bank (Chinese Academy of Sciences, Shanghai, China). The authentication of cells by Short Tandem Repeat (STR) DNA profiling has been done. Monthly *mycoplasma* testing to confirm that cells were not contaminated with *mycoplasma*. A complete endothelial cell medium (ScienCell, CA, United States) was used for HUVECs *in vitro*. Obtain monocyte-derived macrophages (MDM) according to the previous protocol (Menck et al., 2014; Kelly et al., 2018). Briefly, peripheral blood mononuclear cells (PBMC) were purified from human peripheral blood buffy coats by two gradient centrifugations, Mature MDM (M_0_) was then obtained by culturing in 1640 complete medium (BasalMedia, Shanghai, China) containing 50 ng/ml M-CSF for 7 days. Finally, M2 was obtained by polarizing M0 with 20 ng/ml of IL-4 for 48 h. M-CSF (# 300-25) and IL-4 (# 200-04) were purchased from PeproTech (NJ, United States).

### Flow cytometry

The M2 macrophages were separated from the culture plate with cell scrapers and suspended in the staining buffer (1×PBS, 0.1%BSA, 0.1% NaN_3_). Then, non-specific binding was blocked by Human TruStain FcX™ (BioLegend, CA, United States)) for 10 min and then staining with antibodies at 4°C in a dark environment for 30 min. The antibodies used in the process were PE/Dazzle™ 594 anti-human CD200R (OX-108, 1:100) and APC anti-human CD206 (15-2, 1:100), which were purchased from BioLegend (CA, United States). The fluorescence was collected with an ACEA NovoCyte flow cytometer (Agilent, Hangzhou, China), and the data were analyzed and presented by Flowjo 10 (Flowjo, LLC, OR, United States).

### Exosomes isolation and identification

The conditioned culture medium of M2 was collected after 48 h of incubation. Dead cells and large-sized debris were removed by a centrifugation procedure at 2 000 x g for 10 min, followed by a centrifugation procedure at 10 000 x g for 30 min to remove large micro-vesicles. A final exosome-enriched supernatant is collected.

Initial isolation of exosome by PEG precipitation (Ludwig et al., 2018): An equal volume of PEG solution was added to the exosome-enriched solution and mixed thoroughly, PEG buffer containing 20% w/v PEG6000 in 150 mM NaCl, pH 7.0. The mixture was incubated overnight at 4°C and then centrifuged at 1500 g for 30 min for total precipitation. Then the precipitate was resuspended in PBS.

Purification of exosome by size exclusion chromatography (SEC) (Martínez-Greene et al., 2021): For SEC column preparation, Sepharose CL-2B was loaded onto an Econo-Pac chromatography column (Bio-Rad, CA, United States), and the column was washed with 60 ml PBS/0.32% citrate (pH 7.4, 0.22 µm filtration) to remove any ethanol residues from the gel, resulting in a column with a diameter of 1.5 cm and a height of 6.2 cm. 500 μl exosome PBS solution was added to the top of the SEC column, followed by elution with PBS/0.32% citrate. The eluate was collected in 26 consecutive 0.5 ml fractions, and the filtrate from fractions 5-16 was taken and concentrated by ultrafiltration through a 100 kd ultrafilter to give the final purified exosome. PEG6000, Sepharose CL-2B, and citrate were purchased from Sigma-Aldrich (MO, United States).

For the identification of exosomes, the morphology of exosomes was observed by transmission electron microscopy (Hitachi H-7650; Hitachi, Tokyo, Japan); in brief, the exosome products were stained with 2% UO_2_ acetate and photographed and observed by TEM at 80 kV; the particle size distribution of exosomes was analyzed by nanoparticle tracking analysis (Flow NanoAnalyzer, NanoFCM, Xiamen, China). Protein markers such as CD9, CD63, TSG101, and calnexin were detected by Western blot.

### Internalization of exosomes

According to the manufacturer’s instructions, exosomes were labeled with the red fluorescent dye PKH26 (Sigma-Aldrich, MO, United States). The PKH26-labelled exosomes were incubated with HUVECs for 3 h at 37°C in the dark, after which the HUVECs were fixed in 4% paraformaldehyde for 15 min. HUVECs were then treated with 2 mg/ml DAPI for 5 min to stain the nucleus. Finally, they were observed by laser confocal microscopy (Leica SP8, Leica, Wetzlar, Germany) and photographed.

### Cell proliferation assay

Cell counting Kit-8 (absin, Shanghai, China) was used to detect cell proliferation. Proper concentration cells were suspended in 96 wells standard culturing plate. After 8 h of culturing, 10% CCK solution was added to the well, followed by incubating for 40 min. Then the OD 450 nm was collected by a microplate reader.

EdU incorporation was assayed using a Cell-Light EdU Apollo488 *In Vitro* Kit (RiboBio, Guangzhou, China) according to the manufacturer’s instructions. Briefly, Treated or untreated HUVECs were labeled with EdU for 2 h, fixed with 4% paraformaldehyde for 20 min, neutralized with 2 mg/ml glycine for 5 min, permeation with 0.5% TritonX-100 for 10min, stained with Apollo^®^ buffer for 10min at room temperature. EdU incorporation was quantified by measuring the absorbance at 488 nm by a microplate reader (Agilent, Hangzhou, China).

### Wound migration assay

Wound migration assays were carried out by seeding HUVECs into the Culture-Insert 2 Well (ibid; martinsried, Germany). Removed the culture insert after 12 h standard culture, a cell-free gap was created, followed by moving the culture plate into the live-cell working station (Cytation5, BioTek, VT, United States) and setting it to take pictures at specified time points. The wound area was analyzed by ImageJ and calculated according to the equation: migration Rate % = (A_0_ − A_t_)/A_0_ × 100%. A_0_ presented the initial wound area, and A_t_ gave the test wound area.

### Transwell assay

Transwell assay was used to estimate HUVEC migration ability. Briefly, co-culture chambers, purchased from BD Biosciences (CA, United States), were placed in a proper cell culturing plate and divided the well into a top and a bottom region. 1 ml complete medium with 10% fetal serum was added into the base region. Cells suspended within a serum-free medium were evenly added into the top chambers, followed by culturing for 24 h. The non-migration cells were gently removed from the top chambers with a Qtip. The chambers were fixed with 4% methanol for 30 min at room temperature, then stained with crystal violet for another 20 min at room temperature. Before counting stained cells with a microscope, the chambers were washed with PBS and pure water three times and air-dried.

### Tube formation assay

50 μl Matrigel (BD Bioscience, NJ, United States) was added to the 96-well plate, followed by incubating at 37°C for 30 min to form substrate gel. Then, HUVECs from each group were seeded to the substrate gel at a density of 2 × 10^4^/well (three replicates per group). After 6 h incubation at 37°C, tube formation was observed by inverted microscopy (Leica DMI6000B, Wetzlar, Germany). ImageJ measured total branching points and total tube length.

### Cell transfection

The mTOR pathway inhibitor Rapamycin (T1537), purchased from Topscience (Shanghai, China), was dissolved with DMSO in 25 nM. The PI3K/AKT pathway inhibitor, LY294002 (T2008T2008) from (Shanghai, China), was dissolved with DMSO in 50 μM. Incubating time with cells depends on the experiments’ design.

PTEN overexpressed and general control lentivirus was purchased from Genechem (Shanghai, China). The vector was CV186, and the element order was Ubi-MCS-3FLAG-SV40- EGFP -IRES-puromycin. EGFP was employed to validate successful transfection, while puromycin was used to select virus-transfected cells. The transfection process was performed following the standard protocol. Briefly, lentivirus was diluted with the enhanced transfection liquid and polybrene to proper concentration, incubating with cells for 18 h. Subsequently, a fluorescence microscope was employed to validate the successful transfection, and 0.5 mg/ml puromycin was used to filter virus-transfected cells.

The INTERFERin^®^ (Polyplus-transfection^®^ SA, Illkirch, Franch) was used to transfer Cy3-labeled mimics of miR-21 into M2, followed by deriving exosomes from them. These Cy3-mimics-EXO were incubated with HUVECs for 3 h. These HUVECs were fixed in 4% paraformaldehyde and then stained with phalloidin for cytoskeleton and DAPI for nuclei, respectively, followed by observation and photography using fluorescence microscopy. The transfection of miR-21 inhibitor was performed following the same procedure.

### Quantitative reverse transcription-PCR

According to the standard protocol, the total cell RNA solution was extracted with Trizol (Invitrogen, Carlsbad, United States). RNA concentration was validated by Nanodrop (Invitrogen, United States), followed by *in vitro* cDNA synthesis with PrimeScriptTM RT Master Mix kits (Takara, Japan). Then SYBR^®^ Select Master Mix kit (designed for total RNA) was used for RT-qPCR analysis on the Bio‐Rad CFX96 qPCR instrument (Bio‐Rad, Hercules, CA). β-Actin was selected as a reference gene for the coding mRNA test, while U6 was used as a reference gene for the microRNA test. The primers were: PTEN-forward:5′-CAA GAT GAT GTT TGA AAC TAT TCC AAT G-3′; PTEN-Reverse: 5′-CCT TTA GCT GGC AGA CCA CAA-3′; miR-21-5′-GCC CGC TAG CTT ATC AGA CTG ATG-3′; β-actin-F-5′-CTG AAC CCT AAG GCC AAC CG-3′; β-actin-R-5′-GTC ACG CAC GAT TTC CCT CTC-3′; U6-F-5′-GCT TCG GCA GCA CAT ATA CTA AAA T-3′; U6-R-5′-CGC TTC ACG AAT TTG CGT GTC AT-3′.

### Full-thickness cutaneous wounds on mice

All animal experiments were performed following the Guide for the Care and Use of Laboratory Animal (8th edition, Institute of Laboratory Animal Resources, U.S.) and approved by the Animal Care and Experimental Committee of the Faculty of Medicine of Xi’an Jiaotong University.

8-weeks-old male Balb/c mice were obtained from the Medical Laboratory Animal Center of Xi’an Jiao Tong University and randomly separated into 2 groups (20 mice/group). The initial body weights of mice were 20 ± 0.4 g (mean ± 3σ). All mice were housed under specific pathogen-free conditions before and after the operation. Mice were anesthetized with isoflurane, then created a full-thickness cutaneous wound using a 5-mm diameter biopsy punch under an aseptic environment. The M2-EXO solution (200 μg dissolving in 100 μl PBS) or an equal volume of PBS was injected subcutaneously around the wounds, followed by being covered with a skin patch (3 M Health Care, MN, United States). Wounds images were collected by a digital camera on the 0th, 3rd, 7th, and 14th days. ImageJ was used to calculate the wound size. The formula calculated the closure rate of the wounds: Closure rate of the wounds (%) = (C_0_ − C_t_)/C_0_ × 100%. C_0_ was used to present the wound area on the 0th day; Ct was used to represent the wound areas at various time points. At specified time points, the mice were sacrificed with cervical dislocation under anesthetization, then collected 1 cm (Broughton et al., 2006) skin with a sterile instrument and processed according to the subsequent experiments’ design.

### Bioavailability and biosafety testing of exosomes

DIR (Thermo Fisher Scientific; MA, United States) was used to label and visualize M2-EXO for bioavailability assays. DIR-M2-EXO solution (200 μg dissolved in 100 μl PBS) was injected subcutaneously into the mice’s lower back. Fluorescence efficiency of the region of interest (ROI) was collected every 24 h by an *in vivo* imaging system (IVIS) Lumina Series III (PerkinElmer, Massachusetts, United States). Quantifying the fluorescence efficiency was analyzed using Living Image software (PerkinElmer, Massachusetts, United States).

After the experiment, mice blood was collected for a biosafety test. The creatine, BUN, ALT, and AST levels were measured and compared with the reference levels according to the reagent manufacturer’s instructions. The Mouse AST (ab263882), Mouse ALT (ab282882), and Mouse Creatine Kinase (ab285231) were purchased from Abcam (MA, United States). At the same time, the Urea Nitrogen ELISA (EIABUN) kit was bought from Thermo Fisher Scientific (MA, United States).

### Histochemical staining and immunofluorescence analysis

Skin specimens from the 14th day were fixed overnight in 4% paraformaldehyde, dehydrated using serially diluted ethanol, incised pieces individually embedded in paraffin wax, and finally cut into 5 mm sections. Then, wound sections were stained with hematoxylin-eosin (H&E) and Masson’s trichrome staining. Tissue sections were viewed using an inverted microscope (Leica DMI6000B, Wetzlar, Germany) and scanned with a slicing Scanner (Pannoramic DESK, 3DHISTECH, Budapest, Magyarország). For immunofluorescence analysis, paraffin sections were blocked with 1.5% goat serum after dewaxing and antigen repair. Next, sections were incubated with anti-CD31 (1:200) and Anti-alpha smooth muscle Actin (α-SMA, 0.034 μg/ml) overnight at 4°C, followed by Cy3 or FITC conjugated secondary antibody and finally nuclear staining with DAPI (1:50). Tissue sections were observed in a dark environment using a fluorescence microscope (Leica DMI6000B, Wetzlar, Germany) and scanned with slicing Scanner (Pannoramic MIDI, 3DHISTECH, Budapest, Magyarország). Stained tissue sections were analyzed using ImageJ. CD31 (ab76533) and α-SMA (ab7817) were purchased from Abcam (MA, United States). Secondary antibodies were purchased from Jackson Immuno Research (Cambridge, United Kingdom).

The spacing of wound edges in HE sections was measured to determine the wound width, the percentage of collagen stained area in the sarcomeres of Masson sections to determine their collagen volume, the relative thickness of sarcomeres, and the percentage of area stained positively for α-SMA in immunofluorescence sections, the rate of area stained positively for CD31 in sarcomeres, and five random fields of CD31 in each section of sarcomeres were counted and α-SMA double-positive vessels in each tissue section.

### Western blot assay

Lysis buffer, PMSF, and protease inhibitor were purchased from Thermo Fisher Scientific (MA, United States). Protein was extracted from cells or wound tissue following classical protocol. The protein solution was quantified by a BCA Protein Assay Kit (Beyotime, Shanghai, China). The protein samples were diluted to the same concentration with Sample Loading Buffer and used for standard western blot running. Briefly, the protein was added to the running SDS-PAGE gel, and the protein on the gel was transferred to a PVDF membrane (Millipore, Sigma, MO, United States). Subsequently, a 5% BSA-TBST (Sigma, MO, United States) was used for blocking to reduce non-specific binding. After incubating the membrane with the primary antibody overnight, the membrane was washed with TBST and incubated with the secondary antibody for 2 h. After another washing cycle, the membrane was visualized by an ultra-sensitive ECL kit. Results were calculated by Target protein/β-actin based on band intensity tested by ImageJ. The related antibodies used were as follows:

CD9 (ab223052), CD63 (ab216130), TSG101 (ab125011), and calnexin (ab22595) were purchased from Abcam (MA, United States). mTOR (#2983), Phospho-mTOR (Ser2448) (#5536), p70S6K (#9202), Phospho-p70S6K(Thr389) (#9234), PTEN (#9188) were purchased from CST (MA, United States). Phospho-AKT (Ser473) (66444-1-Ig), AKT (10176-2-AP), MMP-2 (10373-2-AP), MMP-9 (10375-2-AP), and β-actin (66009-1-Ig) were purchased from Proteintech (Wuhan, China).

### Enzyme-linked immunoassay

The VEGF ELISA Kits used in this project were all purchased from the R&D system (Human VEGF Quantikine ELISA Kit, DVE00; Mouse VEGF Quantikine ELISA Kit MMV00, Mouse IL-10 Quantikine ELISA Kit M1000B, Mouse IL-1 beta Quantikine ELISA Kit MLB00C, Mouse TNF-alpha Quantikine ELISA Kit MTA00B, and Mouse TGF-beta 1 Quantikine ELISA DB100C).

The whole ELISA process was performed following standard protocol. Briefly, samples were collected from a cell culture medium. 50 μl assay diluent buffer was added to each well, followed by 200 μl of standards, control, or samples per well. After 2 h of incubation, the plate was washed with washing buffer three times, and then the washing buffer was entirely removed by decanting. Subsequently, 200 μl of Detection antibody was added to each well, followed by 2-h room temperature incubation. 200 μl of Substrate Solution was added to each well in a dark environment, and the reaction was stopped by adding 50 μl of Stop Solution after 20 min. The optical density of each well was collected by microplate reader setting to 450 nm.

### Statistics methods

The data in this article are shown as the mean ± 3σ. Data were collected from at least three independent experiments. Paired Student-test was used to test the difference between paired groups. Unpaired Student-test was used to test the difference between un-paired groups. The Chi-square test was used to test the difference for binary variables. Wilcoxon paired test was used to test differences between paired patients’ samples because the difference value does not obey normal distribution. Graphpad (GraphPad Software, La Jolla, CA, United States) was used to analyze the statistics. ImageJ was used to collect intensity from the western blot figure. *p* < 0.05 was marked as *; *p* < 0.01 was marked as **; *p* < 0.005 was marked as***, *p* < 0.001 was marked as ****.

## Results

### The characterization of M2 and exosomes derived from M2

**FIGURE 1:**
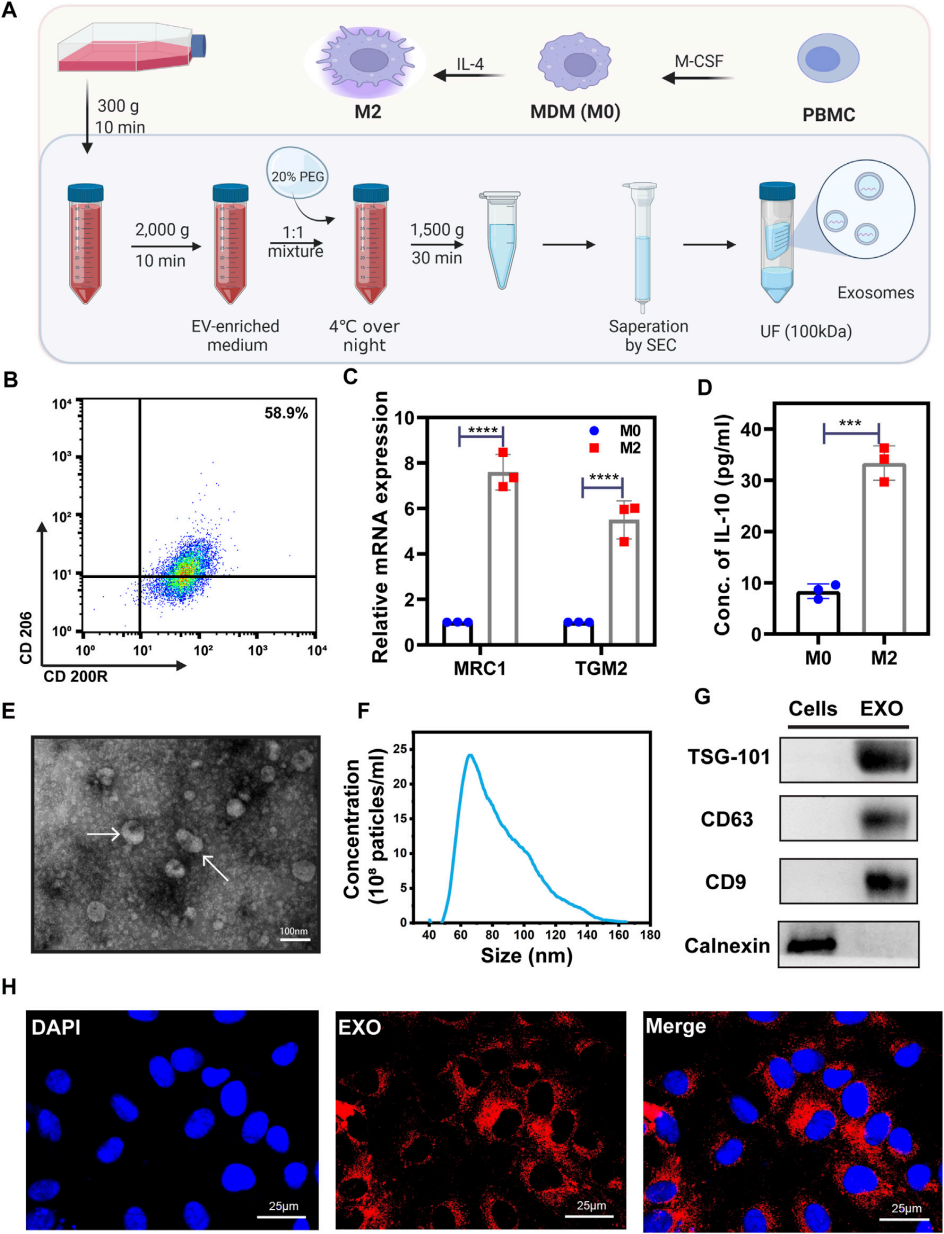
The characterization of M2 and exosome derived from M2. **(A)** Schematic of PEG precipitation exosome extraction flow. A detailed description was shown in the Methods part. Created with BioRender.com. **(B)** Flow cytometry scatter plots of M2 representative surface biomarkers CD200R and CD206 expression level. With a 58.9% CD200R+/CD206+ expression level, these cells were verified as M2 cells. **(C)** qRT-PCR was used to test the expression levels of M2 markers, MRC1, and TGM2. **(D)** ELISA kit was used to test the concentration of IL-10 in the M0 and M2 cell culture medium. **(E)** TEM image of extracted exosomes. Exosomes were labeled by white arrows. **(F)** Nanoparticle Tracking Analysis (NTA) of exosomes. **(G)** Western blot was used to test exosome biomarkers expression. With a higher expression of TSG-101, CD63, CD9, and lower expression of Calnexin compared with normal cells, the extracted vesicles are verified as exosomes. **(H)** Representative immunofluorescence images of PKH26-labeled M2-EXO (red) internalized into HUVECs. Scale bar was shown in the images. Data was expressed as mean ± 3σ (n = 3); * = significant, *p < 0.05; ** *p* < 0.01,***p < 0.005; ****p < 0.001, Student *t*-test.

The M2 macrophages used in our project was induced from PBMC (Figure 1A). To verify the polarization of macrophages, we employed flow cytometry, qRT-PCR, and ELISA to test several characteristic M2 biomarkers, including CD200R, CD206 (MRC1), TGM2, and the secretion of IL-10. The percentage of CD206^+^/CD200R^+^ M2 cells tested by flow cytometry was 58.9% showing a positive result (Figure 1B). After the inducement, the expression of MRC1 (CD206) and TGM2 mRNAs in M2 cells and the IL-10 in M2 culture media significantly increased (Figures 1C,D). These characteristics are consistent with previous studies (Shiratori et al., 2017; Unuvar Purcu et al., 2022), indicating that we successfully obtained M2 macrophages.

We employed PEG precipitation with SEC (Figure 1A) to isolate and purify exosomes from M2 cells (M2-EXOs), and these M2-EXOs were identified by transmission electron microscopy (TEM), nanoparticle tracking analysis (NTA), and western blot. Under the TEM detection field view, M2-EXOs exhibited a classical disc or cup shape (Figure 1E). The extracted exosomes’ range is concentrated around 60–100 nm, an exosome characteristic NTA distribution range (Figure 1F). Moreover, the exosome’s generally positive biomarkers, TSG-101, CD63, and CD9, were positive in our M2-EXOs, while the cellular endoplasmic reticulum-specific protein marker, calnexin, was negative (Figure 1G).

To investigate the M2-EXOs entry feasibility of HUVEC, we performed an *in vitro* tracer experiment. We labeled M2-EXOs with the lipophilic red fluorescent dye PKH26 and incubated them with HUVECs for 3 h, followed by being observed with confocal microscopy. As shown in Figure 1H, the red concentrated in the perinuclear region indicates the internalization of M2-EXOs in HUVEC.

### M2-EXO promotes angiogenesis

**FIGURE 2:**
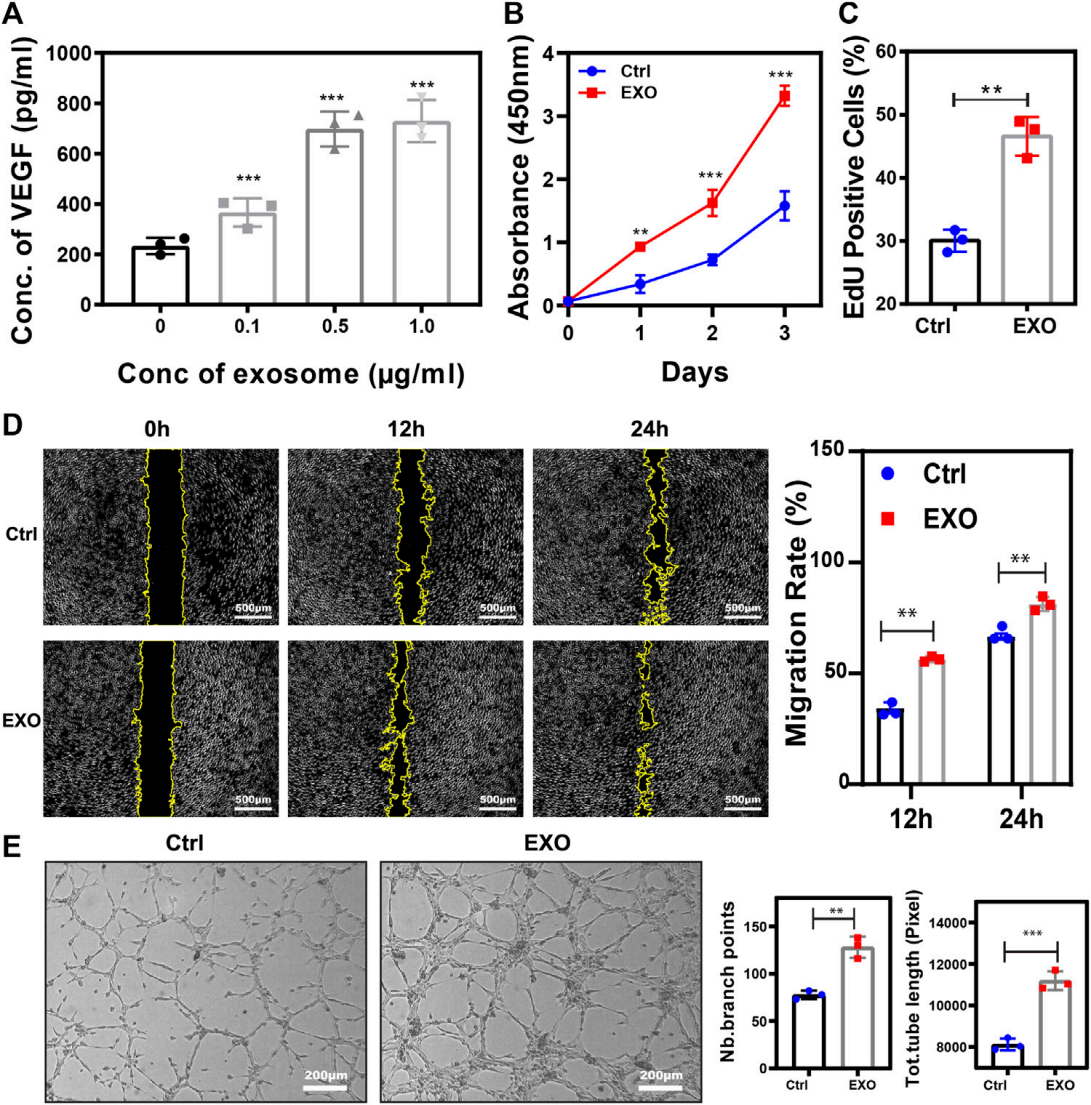
M2-EXO promotes Angiogenesis. **(A)** ELISA assay used to test VEGF concentration in gradient exosomes treated HUVEC culture medium. The VEGF concentration increased with M2-EXO concentration from 0 μg/ml to 1.0 μg/ml and got the optimal concentration in the 0.5 μg/ml group. **(B)** CCK-8 assay was used to estimate HUVEC viability. **(C)** Flow cytometry was used to evaluate the percentage of EdU-positive cells. With a higher EdU positive cell count (46.6 ± 9.2%) than the control group (30.0 ± 5.7%), M2-EXO can increase DNA synthesis, indicating a proliferation improvement. **(D)** Wound healing assay used to test migration ability. Scratch distance and width closure was collected at 0 h, 12 h, and 24 h. The wound area collected at 0 h was qualified and standardized as 100%. After 12 h, the control cells migrated and covered 29.3 ± 3.7% of the wound area, while the exosome incubation cells covered 56.4 ± 3.6% of the wound area. 24 h later, exosomes co-incubated cells migrated 94.5 ± 8.1% of the wound area, significantly higher than the migration area of the control cells. **(E)** Tube formation assay was used to demonstrate the influence of M2-EXO on angiogenesis. ImageJ analyzed the number of branch points and the total tube length. M2-EXO incubated HUVECs had 9992.3 ± 745.8 sizes of entire tube and 76 ± 13.1 branch points, which proved the angiogenesis improvement ability of M2 exosomes. Exosomes derived from M2 was presented as EXO in figures. Data was expressed as mean ± 3σ (*n* = 3); * = significant, *p < 0.05; ** *p* < 0.01,***p < 0.005; ****p < 0.001, Student *t*-test.

Angiogenesis was an essential process in wound healing, and M2 cells were able to promote the process (Mahdavian Delavary et al., 2011). We hypothesized that exosomes derived from M2 played a pivotal modality during angiogenesis promotion. To verify the function of M2-EXO, we test the concentration of VEGF, a commonly recognized angiogenesis indicator (Ferrara, 2004), within different concentrations of M2-EXO treated HUVECs. As shown in Figure 2A, the concentration of VEGF had a dose-dependent increase with M2-EXO, which indicated M2-EXO had a potential for angiogenesis promotion. Meanwhile, we found that 0.5 μg/ml was the optimal M2-EXO working concentration and chose it as the working concentration in the following assays.

To further validate the role of M2-EXO in angiogenesis, we performed several characteristic assays to test the M2-EXOs’ effect on HUVEC viability, proliferation, migration, and tube formation. Firstly, a significantly higher CCK-8 absorbance signal validated viability promotion in the M2-EXO group (Figure 2B). Meanwhile, a higher EdU positive cell percentage in the M2-EXO group than in the control group proved the promotion of proliferation induced by M2-EXO (Figure 2C). Subsequently, we employed a wound migration assay to evaluate the migration level of M2-EXO treated HUVECs. Compared with the control group, the M2-EXO-incubated-HUVEC migrated more of the wound area, indicating a migration promotion role of M2-EXO (Figure 2D). Finally, we performed a tube formation assay on Matrigel, which is recognized as a brilliant mimic of angiogenesis assay *in vitro* (Arnaoutova et al., 2009). As shown in Figure 2E, longer length of the total tube and more branch points proved that M2-EXO treatment increases tube formation in HUVEC cells.

### mTOR signaling participates in angiogenesis promotion induced by M2-EXO

As discussed by recently published research, VEGF signaling was closely associated with mTOR pathway activation and drove angiogenesis (Karali et al., 2014; Oberkersch et al., 2022). Based on the increase of VEGF induced by M2-EXO treatment, we speculated that M2-EXO acted on the mTOR pathway, increasing VEGF expression and angiogenesis.

**FIGURE 3:**
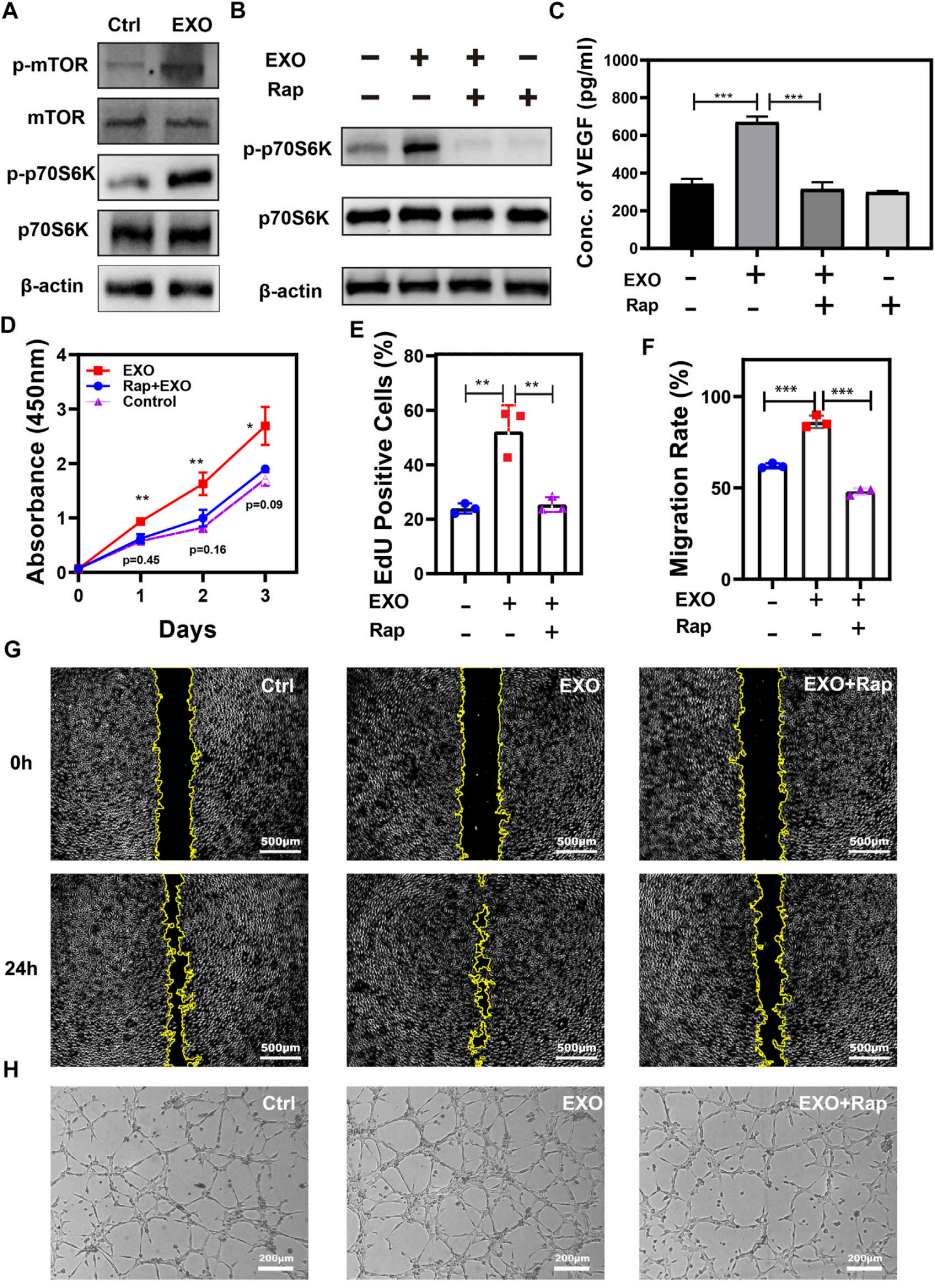
mTOR pathway participates in angiogenesis promotion induced by M2-EXO. **(A)** Western blot was used to test critical markers in the mTOR pathway. Treatment of M2-EXO increased phosphorylation of mTOR and p70S6K. **(B)** Western blot was used to test p-p70S6K expression. Rapamycin treatment counteracted the expression promotion of p-p70S6K induced by M2-EXO. **(C)** ELISA was used to test VEGF expression in Rapamycin treatment HUVEC culture medium. Treatment of Rapamycin counteracted the secretion promotion of VEGF induced by M2-EXO. **(D)** CCK-8 assay was used to demonstrate the promotion role of M2-EXO and the counteraction role of Rapamycin. After M2-EXO treatment, the viability of the cells was sharply increased, while after co-treated with Rapamycin, the viability of the cells was barely equal to the control group. **(E)** Flow cytometry was used to evaluate the percentage of EdU-positive cells. A higher EdU positive cell count (52.3 ± 8.2%) than the control group (23.9 ± 5.6%), while the EdU positive cell counts (25.4 ± 8.1%) of the Rapamycin + M2-EXO were similar to the control group. **(F, G)** The Wound healing assay was used to test migration ability. M2-EXO co-incubated HUVEC migrated the wound area significantly higher than the migration area of the control cells. The Rapamycin + M2-EXO group wound area had no statistical differences from the control group. **(H)** Tube formation assay was used to demonstrate the influence of M2-EXO on angiogenesis. M2-EXO treated group had more lengths of total tube and branch points than the control group, while Rapamycin + M2-EXO treated group had no statistical differences from the control group. Exosomes derived from M2 was presented as EXO in figures. Data was expressed as mean ± 3σ (*n* = 3); * = significant, *p < 0.05; ** *p* < 0.01,***p < 0.005; ****p < 0.001, Student *t*-test.

To assess the role of the mTOR pathway in M2-EXO treatment, we test the expression level of key indicators. We observed phosphorylation promotion of the mTOR and its downstream marker p70S6K (Thr389) in the M2-EXO treatment group, which indicated the activation of the mTOR pathway (Figure 3A). Subsequently, we incubated HUVEC and M2-EXO with or without an mTOR pathway-specific inhibitor, Rapamycin, then tested VEGF expression with ELISA and its results proved that M2-EXO treatment increased VEGF expression. The increasement was fought against by Rapamycin (Figure 3C). The inhibition role of Rapamycin on the mTOR pathway was verified through the expression of p70S6K tested by western blot (Figure 3B).

To further confirm the role of mTOR in M2-EXO-induced angiogenesis, we test the influence of Rapamycin on M2-EXO-induced angiogenic capacity, including proliferation, migration, and tube formation. Priority, we employed EdU incorporation to investigate proliferation capacity, and the results showed that M2-EXO treatment increased the proliferation level of HUVEC, but the co-treatment with rapamycin counteracted this effect (Figures 3D,E). We also assessed the migratory ability of HUVEC with the scratch assay. The migratory area of M2-EXO-treated-HUVEC was nearly twice that of the control group, while the migratory level dropped back in the M2-EXO and rapamycin co-treatment HUVEC (Figures 3F,G). We performed a tubule formation assay of HUVEC on Matrigel. As shown in Figure 3H and Supplementary Figure S1, the treatment of M2-EXO notably increases tube formation, which was proved by the total tube length and branch points. But the addition of rapamycin inhibited the increase. In conclusion, the mTOR’s essential pathway participates in the angiogenesis induced by mTOR.

### M2-EXO activates AKT/mTOR signaling by targeting PETN through miR-21

**FIGURE 4:**
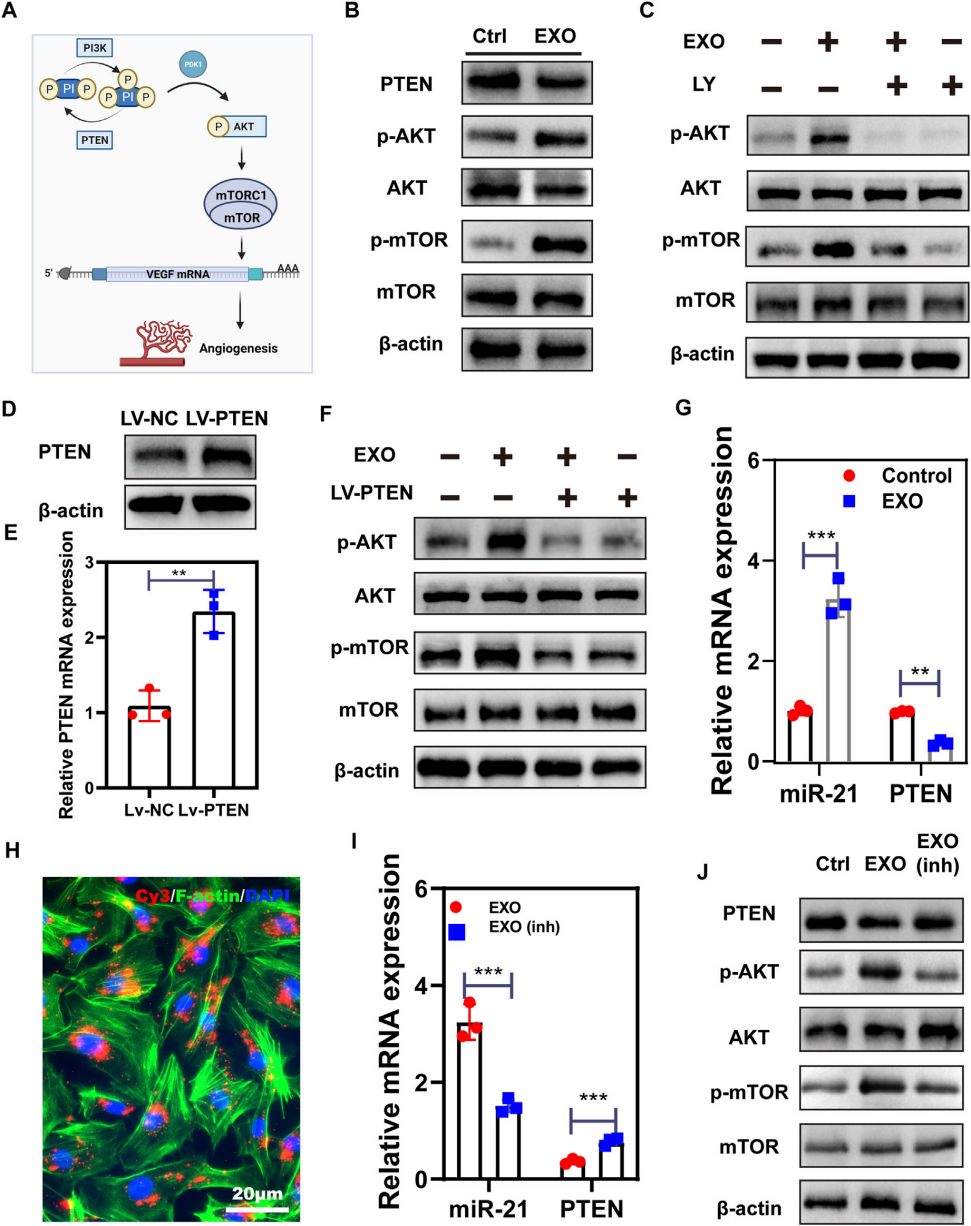
M2-EXO activates AKT/mTOR signaling by targeting PETN through miR-21. **(A)** The schematic of angiogenesis working through the PTEN/AKT/mTOR axis. Created with BioRender.com. **(B)** Western blot was used to test the phosphorylation of PTEN, AKT and mTOR induced by M2-EXO. **(C)** Western blot was used to test the counteraction role of pathway inhibitor LY on phosphorylation of AKT and mTOR induced by M2-EXO. **(D,E)** qRT-PCR and Western blot were used to verify the overexpression of PTEN **(F)** The overexpression of PTEN counteracted the phosphorylation promotion of AKT and the mTOR axis induced by M2-EXO.**(G)** qRT-PCR was used to test miR-21 and PTEN expression levels after M2-EXO treatment. M2-EXO reduced PTEN expression while increased miR-21 expression. **(H)** Florescence of Cy3-miR21-M2-EXO transportation into HUVEC cells, showing vehicle from M2-EXO to HUVECs; **(I)** qRT-PCR was used to test miR-21 and PTEN expression levels after M2-EXO and M2-EXO-miR-21-inhibitor treatment. M2-EXO-inh counteracted the function of M2-EXO. **(J)** Western blot was used to test PTEN and AKT and mTOR phosphorylation after M2-EXO inh treatment. Data was expressed as mean ± 3σ (*n* = 3); * = significant, *p < 0.05; ** *p* < 0.01,***p < 0.005; ****p < 0.001, Student *t*-test.

PI3K/AKT is commonly regarded as an activator of mTOR driving VEGF secretion in the epithelium. Meanwhile, PTEN is a negative indicator of the PI3K/ATK pathway (Karar and Maity, 2011) (Figure 4A). Hence, we employed western blot to test several essential factors in AKT/mTOR. As shown in Figure 4B, the expression of phosphorylated mTOR and AKT notably increased in M2-EXO treated group, while the expression of PTEN decreased. These expression changes suggested that M2-EXO induces Akt activation by inhibiting PTEN expression, further activating the downstream mTOR pathway. To investigate this hypothesis, we first employed LY294002, a commonly used inhibitor of PI3K, to reduce the phosphorylation of AKT. Results showed that pre-treatment with LY294002 attenuated M2-EXO-induced mTOR activation in HUVECs, confirming that M2-EXO activated PI3K/Akt and acted upstream of mTOR (Figure 4C). Subsequently, to verify that M2-EXO activates ATK through targeting PTEN, we built a PTEN overexpressed cell line with lentivirus. We confirmed the successful transfection of PTEN-overexpressed-lentivirus in HUVEC (Figures 4D,E), and then we verified that overexpressed PTEN counteracted the AKT and mTOR activating function of M2-EXO (Figure 4F). This counteraction indicated that M2-EXO activates AKT/mTOR pathway by targeting PTEN.

It has been reported that functional miR-21 is transferred from macrophages to target cells *via* exosomes and acts as a negative regulator of target genes expression (Lan et al., 2019; Chang et al., 2021). To investigate the role of miR-21 in M2-EXO-induced PTEN decrease, we employed qRT-PCR to test miR-21 and PETN expression levels in M2-EXO treated HUVEC. As shown in Figure 4G, M2-EXO treatment increased miR-21 expression and reduced PTEN expression. Subsequently, we transfected Cy3 conjugated miR-21 mimics into M2 macrophages and extracted exosomes from these M2s. We incubated HUVEC with these exosomes and performed an optical test under a fluorescence microscope. We noticed that these HUVEC cells had some red particles, which indicated the transportation of Cy3-labeled-miR-21 mimics (Figure 4H). Besides that, we transfected miR-21 inhibitors into M2, followed by extracting M2-EXO with low expression of miR-21 (M2-EXO inh) (Supplementary Figure S2). Then we co-incubated M2-EXO inh or M2-EXO with HUVEC and tested the expression of PTEN and miR-21. qPCR results show that miR-21-inhibitors confront the miR-21 promotion and PTEN reduction induced by M2-EXO. (Figure 4I). Meanwhile, the western blot also showed that the knockdown of miR-21 rescued PTEN expression levels and caused corresponding changes in AKT and mTOR phosphorylation levels (Figure 4J).

**FIGURE 5:**
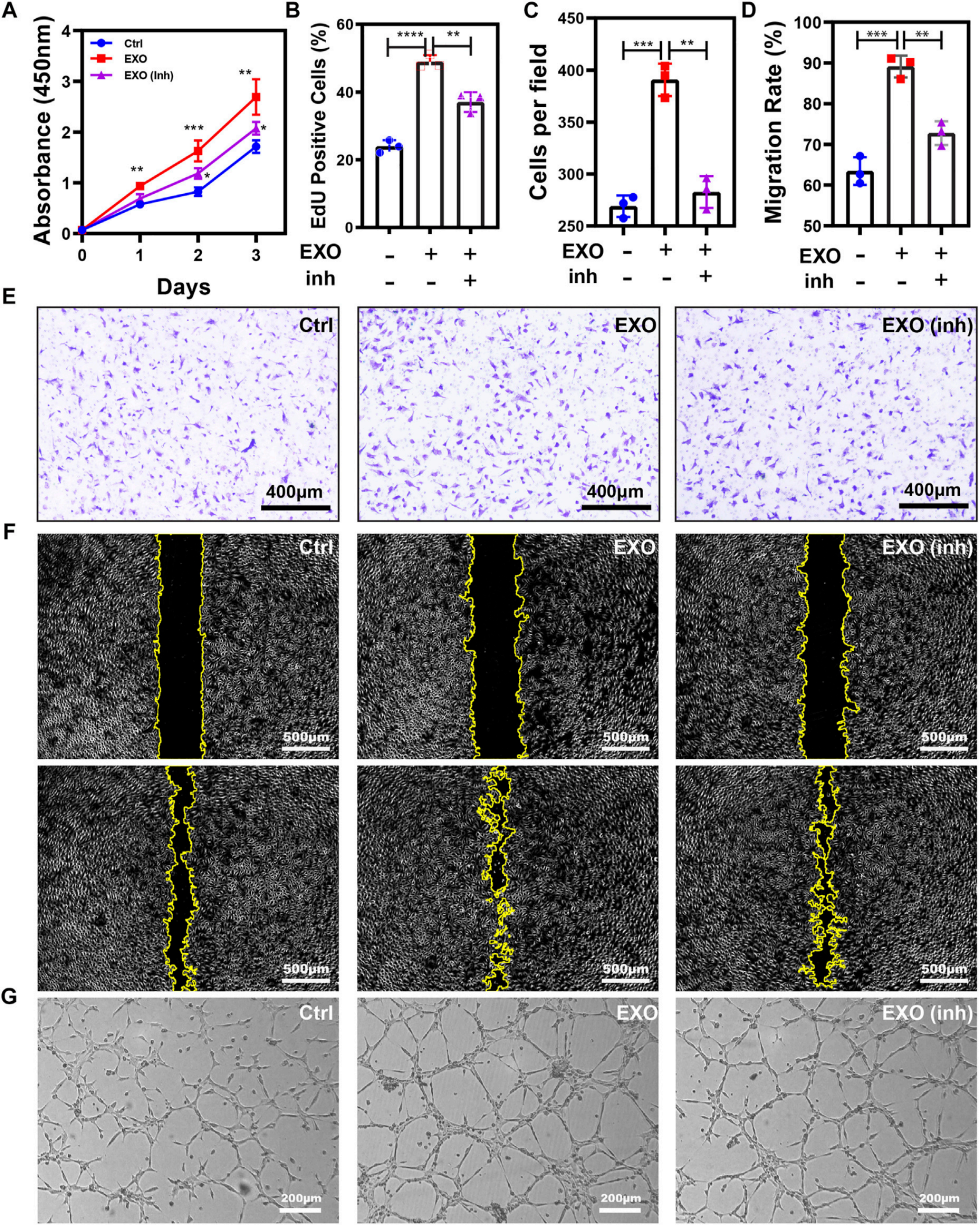
M2-EXO promotes Angiogenesis *via* miR-21. **(A)** CCK-8 assay was used to estimate HUVEC viability. The treatment of M2-EXO significantly increased absorbance at 450nm, while the co-transfection of miR-21 inhibitor counteracted this increasement. The M2-EXO + inhibitor group had slightly higher viability than the control group. **(B)** Flow cytometry was used to evaluate the percentage of EdU-positive cells. The M2-EXO incubated group had a higher EdU-positive cell count than the control group, while the M2-EXO + inhibitor group had a similar EdU-positive cell count to the control group. **(C,E)** Transwell assay was utilized to confirm the metastasis promotion role of M2-EXO treatment visually, and co-transfection of miR-21 inhibitor was used as a rescue factor to confirm M2-EXO works through miR-21. The average number under four fields was regarded as migration cell amounts. **(D,F)** Wound healing assay was used to test migration ability. The M2-EXO + inhibitor group had a higher wound area than the control group but lower than the M2-EXO group, indicating the against the role of miR-21 inhibitor on M2-EXO; **(G)** Tube formation assay was employed to demonstrate the influence of M2-EXO on angiogenesis. M2-EXO incubated HUVECs had longer lengths and more points, while M2-EXO + inhibitor group had similar results to the control group. Data was expressed as mean ± 3σ; (*n* = 3), * = significant, *p < 0.05; ** *p* < 0.01,***p < 0.005; ****p < 0.001, Student *t*-test.

Next, we tested the effect of miR-21 knockdown on angiogenesis induced by M2-EXO. CCK-8 assay and EdU incorporation assay exhibited a proliferation promotion in M2-EXO treated group, while the knockdown of miR-21 counteracted this promotion. (Figures 5A,B). Meanwhile, we employed scratch assay and transwell assay to test the migratory ability of HUVEC. With the knockdown of miR-21, the migration promotion induced by M2-EXO sharply decreased (Figures 5C–F). Similarly, tube formation experiments show similar results (Figure 5G; Supplementary Figure S3). In conclusion, M2-EXO targets PTEN *via* miR-21 to activate AKT/mTOR pathway and promote angiogenesis *in vitro*.

### M2-EXO promotes mice cutaneous wound healing

**FIGURE 6:**
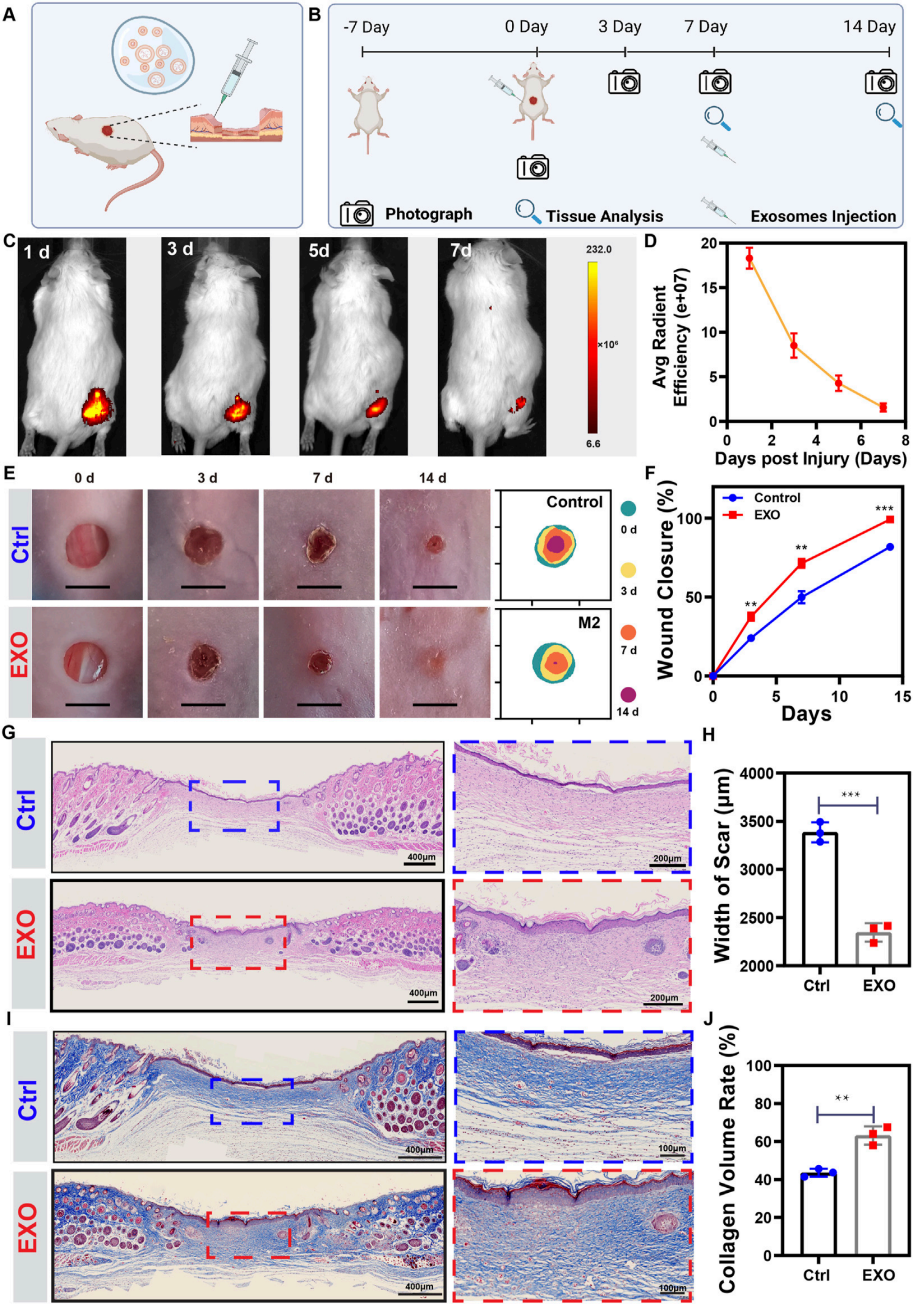
M2-EXO treatment promotes skin wound healing in mice. **(A,B)** Schematic of animal surgery and schedule for animal study. Created with BioRender.com. **(C,D)** IVIS images of Dir-labeled exosomes injected mouse. Images were collected from the same individual mouse. The average fluorescence efficiency was managed and analyzed by Living Image software. (*n* = 4). **(E,F)** The gross view and healing trace of the wound in control and M2-EXO treated groups. Images and healing traces were collected from the same mouse randomly chosen from the control and the M2-EXO treated group. The wound closure rate was analyzed according to the healing trace. (*n* = 3). **(G)** Representative figures of HE staining wound. Scale bar was labeled in the figure. **(H)** Width of scar. (*n* = 3). **(I)** Representative figures of Masson staining wound. Scale bar was labeled in the figure. **(J)** Quantitative analysis of collagen volume in each group. (*n* = 3). Data was expressed as mean ± 3σ; * = significant, *p < 0.05; **p < 0.01,***p < 0.005; ****p < 0.001, Student *t*-test.

Angiogenesis is essential for wound healing (Noishiki et al., 2019). To assess the influence of M2-EXO to wound healing, we constructed a 5 mm diameter full-thickness cutaneous wound on mice’s back, followed by injecting PBS solution with or without M2-EXO around the wound area. The schematic was shown in Figure 6A. IVIS real-time imaging system showed that the exosomes labeled with DIR had significant fluorescence signals in the subcutaneous tissue, which gradually degraded over 7 days to less than 10% (Figures 6C,D). Due to the degradation rate, we injected M2-EXO solution into the wound site every 7 days. The experimental design of the animal study was shown in Figure 6B. According to the wound closure images at each time point, the M2-EXO group had a faster healing rate than the control group. As shown in Figures 6E,F, on the 14th day, the wound of the M2-EXO group was healed entirely, while the damage of the control group only healed around 81.85 ± 6.62%. The promotion of wound healing was most likely attributed to the positive effect of M2-EXO on angiogenesis.

To accurately evaluate the wound healing status, we performed histochemical staining with the wound tissue on the 14th day. Hematoxylin and eosin (H&E) staining showed that M2-EXOs-treated wounds exhibited a better level of tissue regeneration, shorter width of scar, and thicker and denser granulation tissue with regenerated skin attachments such as hair follicles and glands, which indicated that M2-EXOs accelerated the skin regeneration process (Figures 6G,H). Masson staining showed that the M2-EXO treated mice had more mature collagen fibers and more extensive collagen deposition than the control group, indicating a higher ECM remodeling level (Figures 6I,J). Besides, we tested renal function indicators (creatine and BUN) and liver function indicators (ALT and AST) to assess the health status of the mice. With no abnormalities, these results confirmed the biosafety of exosome treatment (Supplementary Figure S4).

### M2-EXO treatment promotes angiogenesis and regeneration in wound

**FIGURE 7:**
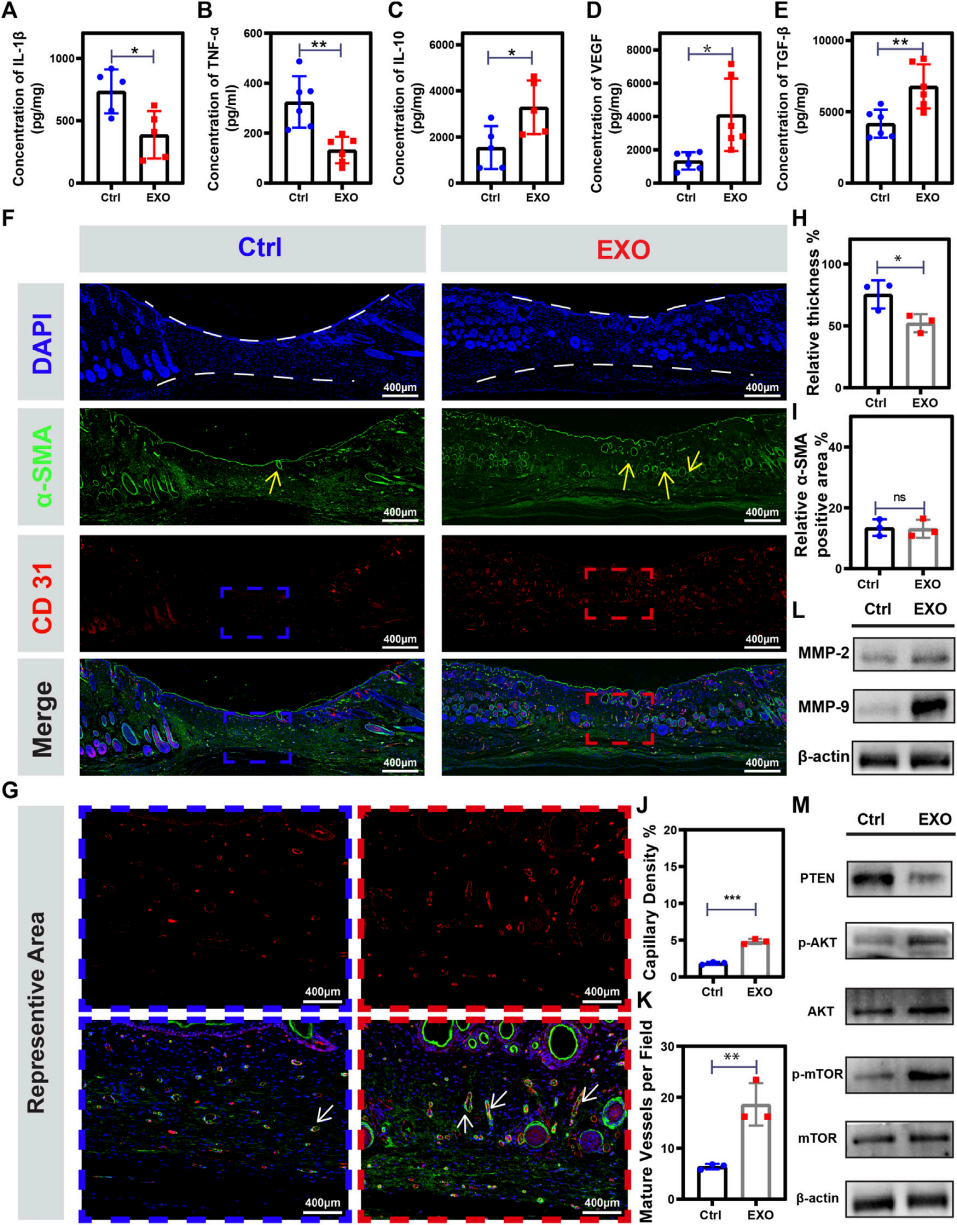
M2-EXO treatment promotes angiogenesis and tissue regeneration in the wound. **(A–E)** ELISA assay used to test TNF-α, IL-1β, IL-10, TGF-β, and VEGF concentration in on the 7th day. (*n* = 6). **(F)** Immune-fluorescence images were collected from tissue homogenization on the 14th day from the control group, and the M2-EXO treated group, respectively. Red dye was used to label CD31; Green dye was used to label α-SMA; DAPI was used to label the nucleus; Merge figure was presented by integrating all the figures. Scale bar was labeled in the figures. **(G)** Representative immunofluorescence figures selected from **(F)** followed by being enlarged. **(H)** Quantification analysis of the relative thickness of granulation tissue (*n* = 3). **(I)** Quantification analysis of α-SMA-positive staining area (*n* = 3) **(J)** Quantification analysis of vessel density in sarcomeres (*n* = 3) **(K)** Quantification analysis of the number of mature blood vessels in sarcomeres (*n* = 3). **(L**–**M)** Western blot was used to test the expression level of MMP-2, MMP-9, PTEN, p-AKT, AKT, mTOR, and p-mTOR in tissue homogenization collected on the 7th day. Data was expressed as mean ± 3σ; * = significant, *p < 0.05; ** *p* < 0.01,***p < 0.005; ****p < 0.001, Student *t*-test.

The interconversion expression between pro-inflammatory and anti-inflammatory cytokines was a critical regulator of wound healing. Hence, we employed ELISA to analyze the expression level of pro-inflammatory cytokines (IL-1β and TNF-α) and anti-inflammatory cytokines (IL-10) on the 7th day. ELISA results showed a significantly lower expression of IL-1β and TNF-α and higher expression of IL-10 in the M2-EXO treated mouse wounds than in the control mouse (Figures 7A–C), which indicated treatment with M2-EXO significantly promoted the conversation from pro-inflammatory to anti-inflammatory expression in wound tissue. Then, we tested the expression of growth factors (TGF- β, and VEGF) to evaluate tissue repair and regeneration levels. Compared with the control mouse, the wound tissue of the M2-EXO treated mouse has a high level of TGF- β, and VEGF. These results suggested that M2-EXO treatment promotes an anti-inflammatory phenotype conversion in mouse wound, increases growth factors TGF-β and VEGF expression, and accelerates wound healing. (Figures 7D,E). High levels of growth factors indicate high levels of tissue regeneration. To assess tissue repair and regeneration levels, we quantified the relative thickness of granulation tissue and employed it as an indicator of tissue regeneration. We found that M2-EXO-treated mice showed greater relative thickness of granulation tissue than control mice on the 7th day (Figures 7F,H). Meanwhile, α-SMA, a marker of myofibroblasts, can reflect the maturation level of granulation tissue. The immunofluorescence images showed that the positive α-SMA percentage exhibited no statistical differences between M2-EXO treated group and the control group. (Figures 7F,I). However, the α-SMA positive region in the M2-EXO group was mainly located in the skin functional structures such as hair follicles, glands, and blood vessels, which were largely absent in the control group (Figure 7F, α-SMA part, labeled by yellow arrows). These results proved a higher level of granulation tissue maturation in the M2-EXO treated group, which indicated that the treatment of M2-EXO reduced myofibroblasts in middle and late wound healing and prevented hyper-fibrosis development and scar formation, which is also consistent with our observation of less scar formation in M2-EXO-treated mice.

The vascularization process was essential for complex and dynamic wound healing [ref]. CD31 was a vascular endothelial cells indicator and cooperated with α-SMA to localize mature vessels. We found the wound tissues of M2-EXO treated mice had a higher level of vascularization with higher vessel density and more mature vessels than the wound tissues of control mice (Figures 7G,J,K). Besides, results of western blot angiogenesis-related Metallo-matrix proteases, MMP-9 and MMP-2, illustrated a higher expression in M2-EXO treated mice (Figure 7L). These results suggested treatment of M2-EXO promoted the vascularization process. We have confirmed M2-EXO working through targeting PETN to promote phosphorylation of AKT and mTOR *in vitro*. Subsequently, we aim to demonstrate the gene regulator role of M2-EXO *in vivo*. Consistent with the results in HUVEC cells, p-AKT and p-mTOR significantly increased in the 7th mice wound tissue (Figure 7M). These results indicated that treatment of M2-EXO promoted angiogenesis in mice tissue and played a beneficial role in skin repair by improving the speed and quality of wound healing.

## Discussion

The angiogenesis promotion role of M2 macrophages has been a consensus (Sunderkötter et al., 1991; Jetten et al., 2014), and studies about the relationship between M2 and angiogenesis have caught researchers’ attention. M2 promotes angiogenesis in several ways (Ngambenjawong et al., 2017; Ueta et al., 2019; Zhang et al., 2020), and the exosomes derived from M2 have broadly been regarded as an essential angiogenesis promotion (Gangadaran et al., 2020; Luo et al., 2021; Yang et al., 2021). However, the exact mechanisms of angiogenesis promotion induced by M2 are still unclear. In our project, we found that M2-EXO target PTEN *via* miR-21 to activate AKT/mTOR pathway and promote angiogenesis *in vitro*. Besides, we employed a whole skin wounding mouse model to demonstrate that the treatment of M2-EXO significantly promotes angiogenesis and accelerates the wound healing process, proving the potential of M2-EXO therapy in repairing skin injury.

VEGF is one of the most effective angiogenesis promotion factors and an essential indicator of angiogenesis (Ferrara, 2004). To test the function of M2-EXO on VEGF secretion, we used a VEGF ELIS kit to test the culture medium of HUVEC. We found that M2-EXO promoted VEGF secretion in a dose-dependent way, which strongly suggested the positive role of M2-EXO on angiogenesis. Subsequently, we tested proliferation, migration, and tube formation ability with CCK-8, Edu, wound healing, and tube formation assays. The results of these assays clarified the angiogenesis-promoting effect of M2-EXO *in vitro*. Then, we investigated the molecular mechanism of angiogenesis stimulated by M2-EXO *in vitro*. mTOR is a highly stable serine/threonine-protein kinase that plays an essential role in cell proliferation and metabolism (Liu and Sabatini, 2020). Activated mTOR phosphorylates the downstream factors S6K1 4EBP1 and STAT3, followed by synthesizing transcription factors, such as HIF-1 α, which leads to a high expression of VEGF and promotes angiogenesis (Dodd et al., 2015). We investigated the M2-EXO treatment leaded phosphorylation of S6K1 at the Thr-389 site, indicating the activation of the mTOR pathway after M2-EXO treatment. Rapamycin worked as an mTOR pathway inhibitor by forming a complex with the endogenous protein FKBP (Yang et al., 2013). We employed Rapamycin as a rescue factor in our project, and the results showed that treatment of Rapamycin counteracted the VEGF expression increase induced by M2-EXO. These results fully explained the role of the mTOR pathway in angiogenesis promotion induced by M2-EXO treatment. PI3K-AKT is an essential mitogenic signaling pathway closely associated with cell growth, proliferation, survival, and motility (Somanath et al., 2008; Karar and Maity, 2011). Meanwhile, PI3K promotes the conversion of PtdIns (4,5) P2 to PtdIns (3,4,5) P3, and PtdIns (3,4) P2. While PtdIns (3,4,5) P3 and PtdIns (3,4) P2 are able to directly bind with AKT on PH structural domain, followed by recruiting PDK1 to the plasma membrane and promoting AKT phosphorylation on the Thr308 site, which activated downstream mTOR and other signals (Primo et al., 2007). In contrast, PTEN negatively regulates the PI3K-AKT/mTOR pathway by converting PtdIns (3,4,5) P3 to PtdIns (4,5) P2 to antagonize PI3K (Song et al., 2012). In this study, HUVECs treated with LY294002 + M2-EXO had a lower mTOR level activation than the M2-EXO individually treated group, suggesting that PI3K/Akt acts upstream of mTOR. Meanwhile, the overexpression of PTEN also counteracted the activation of AKT/mTOR, which indicated that M2-EXO activates Akt/mTOR signaling by targeting PTEN.

Our next aim was to reveal the inhibition mechanism of PTEN expression induced by M2-EXO. Numerous studies have shown that the most biological effects of exosomes are attributed to their translocated miRNAs (Zhang et al., 2015). Some publications have reported that exosomal-miR-21 transfers from macrophages to target cells followed by targeting PTEN (Zheng et al., 2017). Meanwhile, some fascinating studies have identified the role of miR-21 in regulating angiogenesis, EC survival, and vascular remodeling (Canfrán-Duque et al., 2017; Spinetti et al., 2020; Liao et al., 2021). Besides, exosomes derived from umbilical cord blood mesenchymal stem cells and amniotic fluid stem cells are able to promote skin regeneration by carrying miR-21 (Zhang et al., 2021a; Zhang et al., 2021b). Therefore, it is reasonable to hypothesize that M2-EXO promotes angiogenesis by transferring miR-21 into HUVEC and targeting PTEN to activate the AKT/mTOR pathway. Our study found that M2-EXO transferred miR-21 into HUVEC, inhibiting PTEN mRNA expression. In contrast, the exosomes derived from miR-21 inhibiting M2 reduced the transfection of miR-21 to HUVEC and then recovered PTEN mRNA expression, while the phosphorylation levels of AKT and mTOR showed a corresponding decrease. Additional characteristic experiments of proliferation, migration, and tube formation also exhibited that M2-EXO-associated miR-21 promoted angiogenesis.

Poor postoperative and post-trauma healing influences millions of people worldwide (Kim et al., 2019), and the acceleration of the wound healing process is critical to reconstructing damaged skin tissue and restoring the integrity of skin structure and function (Sorg et al., 2017). M2 macrophages, the vital cells for wound healing, is able to promote migration of epithelial cells, angiogenesis, and proliferation of fibroblasts to further improve wound healing. But the role of exosomes derived from M2 macrophages in wound healing has not been clarified. Our project showed that M2-EXO was highly effective in promoting wound healing, confirmed by observations of wound appearance and wound length measurements in tissue sections. Quality is another crucial factor beyond the speed when evaluating the wound healing process (Kim et al., 2019). The microstructure of the wound tissue exhibited that M2-EXO-treated wounds had a better level of tissue regeneration than PBS-treated control wounds. We observed thicker and denser granulation tissue and more extensive mature collagen deposition in the M2-EXO treated group and found skin attachment structures such as hair follicles and glands in their granulation tissue, with a low level of inflammation facilitated tissue remodeling (Sindrilaru and Scharffetter-Kochanek, 2013; Hesketh et al., 2017). Besides, myofibroblasts in wound granulation tissue are closely associated with scar formation (Pakshir et al., 2020). The wound tissue treated by M2-EXO had fewer myofibroblasts, which explained the smoother skin wound appearance in the M2-EXO treatment group and a scar tendency in the control group. Overall, M2-EXO treatment significantly promoted high-quality skin healing, which could be attributed to the angiogenesis-promoting effect of M2-EXO. Wound healing is an orderly and complex process of tissue regeneration, and angiogenesis is present throughout (Veith et al., 2019). Because only the transport of building materials involving cells, growth factors, and oxygen by new blood vessels to the reconstructed wound site can ensure continuous and orderly tissue regeneration (Arnold and West, 1991). In this study, M2-EXO administration produced many CD31-positive vessels in the wound area, which mechanistically could be since M2-EXO induced the proliferation, survival, and migration of vascular endothelial cells through activation of the PTEN/AKT/mTOR pathway and thus promoting massive angiogenesis. We also noted that the M2-EXO-treated group showed increased expression of TGF-β, VEGF, MMP2, and MMP-9. TGF-β and VEGF are significant for granulation tissue formation and vascularization (Barrientos et al., 2008; Behm et al., 2012), while MMP-2 and MMP-9 (the MMPs related to angiogenesis) are also important for ECM remodeling (van Hinsbergh et al., 2006; Quintero-Fabián et al., 2019). Therefore, we suggested that M2-EXO created positive conditions for angiogenesis in wound tissue at the cellular level and the extracellular matrix microenvironment, thus promoting the healing process with high efficiency and quality and playing an active role in the repair and regeneration of skin wounds.

The exosome treatment, a cell-free therapy, has emerged as a promising nanomedicine strategy with low immunogenicity, high stability, and easy clinical translation (Kalluri and LeBleu, 2020; Aslan et al., 2021; Lin et al., 2022). Except for wound healing, exosome therapy can be employed in treating other ischemic diseases involving peripheral arterial disease, coronary artery disease, bone defects, and osteonecrosis (Ibrahim and Marbán, 2016; Shan et al., 2019; Bei et al., 2021). Our findings suggested that M2-EXO therapy had reliable applications in the treatment of skin tissue regeneration. With lots of reliable results, our project was limited by the differences in pathophysiology between rodents and human skin (Lindblad, 2008). Hence, our further task would be to conduct experiments on animals with closer anatomy and physiology, such as swine (Swindle et al., 2012), followed by optimizing the treatment protocol, including adjusting the dose and timing of administration. Besides, the model constructed in this project is an acute wound healing model (Masson-Meyers et al., 2020), while more efforts should be targeted to chronically healing wounds, such as diabetic or aging skin wounds (Wilkinson and Hardman, 2020). These chronic wounds will be another direction to explore the M2-EXO therapy function further.

## Data Availability

The original contributions presented in the study are included in the article/Supplementary Material, further inquiries can be directed to the corresponding authors.

## Abbreviations

ALT, alanine transaminase; AST, aspartate aminotransferase; BUN, Blood urea nitrogen; Ctrl, Control; EXO; exosome derived from M2 macrophages; Inh, inhibitor; LY, LY294002; M2, Type 2 macrophages; Rap, Rapacymin.

## References

[B1] An Y. Lin S. Tan X. Zhu S. Nie F. Zhen Y. (2021). Exosomes from adipose-derived stem cells and application to skin wound healing. Cell Prolif. 54 (3), e12993. 10.1111/cpr.12993 33458899 PMC7941238

[B2] Arnaoutova I. George J. Kleinman H. K. Benton G. (2009). The endothelial cell Tube formation assay on basement membrane turns 20: state of the science and the art. Angiogenesis 12 (3), 267–274. 10.1007/s10456-009-9146-4 19399631

[B3] Arnold F. West D. C. (1991). Angiogenesis in wound healing. Pharmacol. Ther. 52 (3), 407–422. 10.1016/0163-7258(91)90034-j 1726477

[B4] Aslan C. Kiaie S. H. Zolbanin N. M. Lotfinejad P. Ramezani R. Kashanchi F. (2021). Exosomes for mRNA delivery: a novel biotherapeutic strategy with hurdles and hope. BMC Biotechnol. 21 (1), 20. 10.1186/s12896-021-00683-w 33691652 PMC7945253

[B5] Barrientos S. Stojadinovic O. Golinko M. S. Brem H. Tomic-Canic M. (2008). Growth factors and cytokines in wound healing. Wound Repair Regen. 16 (5), 585–601. 10.1111/j.1524-475X.2008.00410.x 19128254

[B6] Behm B. Babilas P. Landthaler M. Schreml S. (2012). Cytokines, chemokines and growth factors in wound healing. J. Eur. Acad. Dermatol. Venereol. 26 (7), 812–820. 10.1111/j.1468-3083.2011.04415.x 22211801

[B7] Bei H. P. Hung P. M. Yeung H. L. Wang S. Zhao X. (2021). Bone-a-Petite: Engineering exosomes towards bone, osteochondral, and cartilage repair. Small (Weinheim der Bergstrasse, Ger. 17 (50), e2101741. 10.1002/smll.202101741 34288410

[B8] Broughton G. 2nd Janis J. E. Attinger C. E. (2006). Wound healing: an overview. Plast. Reconstr. Surg. 117 (7), 1e-S–32e-S. 10.1097/01.prs.0000222562.60260.f9 16801750

[B9] Canfrán-Duque A. Rotllan N. Zhang X. Fernandez-Fuertes M. Ramirez-Hidalgo C. Araldi E. (2017). Macrophage deficiency of miR-21 promotes apoptosis, plaque necrosis, and vascular inflammation during atherogenesis. EMBO Mol. Med. 9 (9), 1244–1262. 10.15252/emmm.201607492 28674080 PMC5582411

[B10] Chang J. Li H. Zhu Z. Mei P. Hu W. Xiong X. (2021). microRNA-21-5p from M2 macrophage-derived extracellular vesicles promotes the differentiation and activity of pancreatic cancer stem cells by mediating KLF3. Cell Biol. Toxicol. 38, 577–590. 10.1007/s10565-021-09597-x 33728488 PMC9343318

[B11] Chin J. S. Madden L. Chew S. Y. Becker D. L. (2019). Drug therapies and delivery mechanisms to treat perturbed skin wound healing. Adv. Drug Deliv. Rev. 149-150, 2–18. 10.1016/j.addr.2019.03.006 30959068

[B12] Colombo M. Raposo G. Théry C. (2014). Biogenesis, secretion, and intercellular interactions of exosomes and other extracellular vesicles. Annu. Rev. Cell Dev. Biol. 30, 255–289. 10.1146/annurev-cellbio-101512-122326 25288114

[B13] Dodd K. M. Yang J. Shen M. H. Sampson J. R. Tee A. R. (2015). mTORC1 drives HIF-1α and VEGF-A signalling via multiple mechanisms involving 4E-BP1, S6K1 and STAT3. Oncogene 34 (17), 2239–2250. 10.1038/onc.2014.164 24931163 PMC4172452

[B14] Ferrara N. (2004). Vascular endothelial growth factor: basic science and clinical progress. Endocr. Rev. 25 (4), 581–611. 10.1210/er.2003-0027 15294883

[B15] Gangadaran P. Rajendran R. L. Oh J. M. Hong C. M. Jeong S. Y. Lee S. W. (2020). Extracellular vesicles derived from macrophage promote angiogenesis *in vitro* and accelerate new vasculature formation *in vivo* . Exp. Cell Res. 394 (2), 112146. 10.1016/j.yexcr.2020.112146 32561287

[B16] Gravitz L. (2018). Skin. Nature 563 (7732), S83. 10.1038/d41586-018-07428-4 30464282

[B17] Greaves N. S. Lqbal S. A. Morris J. Benatar B. Alonso-Rasgado T. Baguneid M. (2015). Acute cutaneous wounds treated with human decellularised dermis show enhanced angiogenesis during healing. PloS one. 10 (1), e0113209. 10.1371/journal.pone.0113209 25602294 PMC4300088

[B18] Gurevich D. B. Severn C. E. Twomey C. Greenhough A. Cash J. Toye A. M. (2018). Live imaging of wound angiogenesis reveals macrophage orchestrated vessel sprouting and regression. EMBO J. 37 (13), e97786. 10.15252/embj.201797786 29866703 PMC6028026

[B19] Hesketh M. Sahin K. B. West Z. E. Murray R. Z. (2017). Macrophage phenotypes regulate scar formation and chronic wound healing. Int. J. Mol. Sci. 18 (7), E1545. 10.3390/ijms18071545 28714933 PMC5536033

[B20] Hettich B. F. Ben-Yehuda Greenwald M. Werner S. Leroux J. C. (2020). Exosomes for wound healing: Purification optimization and identification of bioactive components. Adv. Sci. 7 (23), 2002596. 10.1002/advs.202002596 PMC7709981 33304765

[B21] Hu Y. Rao S. S. Wang Z. X. Cao J. Tan Y. J. Luo J. (2018). Exosomes from human umbilical cord blood accelerate cutaneous wound healing through miR-21-3p-mediated promotion of angiogenesis and fibroblast function. Theranostics. 8 (1), 169–184. 10.7150/thno.21234 29290800 PMC5743467

[B22] Ibrahim A. Marbán E. (2016). Exosomes: Fundamental biology and roles in cardiovascular physiology. Annu. Rev. Physiol. 78, 67–83. 10.1146/annurev-physiol-021115-104929 26667071 PMC5425157

[B23] Jetten N. Verbruggen S. Gijbels M. J. Post M. J. De Winther M. P. Donners M. M. (2014). Anti-inflammatory M2, but not pro-inflammatory M1 macrophages promote angiogenesis *in vivo* . Angiogenesis. 17 (1), 109–118. 10.1007/s10456-013-9381-6 24013945

[B24] Kalluri R. LeBleu V. S. (2020). The biology, function, and biomedical applications of exosomes. Science. 367, eaau6977. 10.1126/science.aau6977 32029601 PMC7717626

[B25] Karali E. Bellou S. Stellas D. Klinakis A. Murphy C. Fotsis T. (2014). VEGF signaling, mTOR complexes, and the endoplasmic reticulum: Towards a role of metabolic sensing in the regulation of angiogenesis. Mol. Cell. Oncol. 1 (3), e964024. 10.4161/23723548.2014.964024 27308350 PMC4904886

[B26] Karar J. Maity A. (2011). PI3K/AKT/mTOR pathway in angiogenesis. Front. Mol. Neurosci. 4, 51. 10.3389/fnmol.2011.00051 22144946 PMC3228996

[B27] Kelly A. Grabiec A. M. Travis M. A. (2018). Culture of human monocyte-derived macrophages. Methods Mol. Biol. 1784, 1–11. 10.1007/978-1-4939-7837-3_1 29761383

[B28] Kim H. S. Sun X. Lee J. H. Kim H. W. Fu X. Leong K. W. (2019). Advanced drug delivery systems and artificial skin grafts for skin wound healing. Adv. Drug Deliv. Rev. 146, 209–239. 10.1016/j.addr.2018.12.014 30605737

[B29] Lan J. Sun L. Xu F. Liu L. Hu F. Song D. (2019). M2 macrophage-derived exosomes promote cell migration and invasion in colon cancer. Cancer Res. 79 (1), 146–158. 10.1158/0008-5472.CAN-18-0014 30401711

[B30] Liao Z. Chen Y. Duan C. Zhu K. Huang R. Zhao H. (2021). Cardiac telocytes inhibit cardiac microvascular endothelial cell apoptosis through exosomal miRNA-21-5p-targeted cdip1 silencing to improve angiogenesis following myocardial infarction. Theranostics. 11 (1), 268–291. 10.7150/thno.47021 33391474 PMC7681094

[B31] Lin Y. Y. Chen C. Y. Ma D. L. Leung C. H. Chang C. Y. Wang H. D. (2022). Cell-derived artificial nanovesicle as a drug delivery system for malignant melanoma treatment. Biomed. Pharmacother. = Biomedecine Pharmacother. 147, 112586. 10.1016/j.biopha.2021.112586 34999373

[B32] Lindblad W. J. (2008). Considerations for selecting the correct animal model for dermal wound-healing studies. J. Biomater. Sci. Polym. Ed. 19 (8), 1087–1096. 10.1163/156856208784909390 18644233

[B33] Liu G. Y. Sabatini D. M. (2020). mTOR at the nexus of nutrition, growth, ageing and disease. Nat. Rev. Mol. Cell Biol. 21 (4), 183–203. 10.1038/s41580-019-0199-y 31937935 PMC7102936

[B34] Ludwig A. K. De Miroschedji K. Doeppner T. R. Borger V. Ruesing J. Rebmann V. (2018). Precipitation with polyethylene glycol followed by washing and pelleting by ultracentrifugation enriches extracellular vesicles from tissue culture supernatants in small and large scales. J. Extracell. Vesicles. 7 (1), 1528109. 10.1080/20013078.2018.1528109 30357008 PMC6197019

[B35] Luo Z. Peng W. Xu Y. Xie Y. Liu Y. Lu H. (2021). Exosomal OTULIN from M2 macrophages promotes the recovery of spinal cord injuries via stimulating Wnt/β-catenin pathway-mediated vascular regeneration. Acta Biomater. 136, 519–532. 10.1016/j.actbio.2021.09.026 34551329

[B36] Mahdavian Delavary B. van der Veer W. M. van Egmond M. Niessen F. B. Beelen R. H. (2011). Macrophages in skin injury and repair. Immunobiology. 216 (7), 753–762. 10.1016/j.imbio.2011.01.001 21281986

[B37] Martínez-Greene J. A. Hernández-Ortega K. Quiroz-Baez R. Resendis-Antonio O. Pichardo-Casas I. Sinclair D. A. (2021). Quantitative proteomic analysis of extracellular vesicle subgroups isolated by an optimized method combining polymer-based precipitation and size exclusion chromatography. J. Extracell. Vesicles. 10 (6), e12087. 10.1002/jev2.12087 33936570 PMC8077108

[B38] Masson-Meyers D. S. Andrade T. A. M. Caetano G. F. Guimaraes F. R. Leite M. N. Leite S. N. (2020). Experimental models and methods for cutaneous wound healing assessment. Int. J. Exp. Pathol. 101 (1-2), 21–37. 10.1111/iep.12346 32227524 PMC7306904

[B39] Menck K. Behme D. Pantke M. Reiling N. Binder C. Pukrop T. (2014). Isolation of human monocytes by double gradient centrifugation and their differentiation to macrophages in teflon-coated cell culture bags. J. Vis. Exp. (91), e51554. 10.3791/51554 25226391 PMC4828059

[B40] Morel O. Toti F. Hugel B. Bakouboula B. Camoin-Jau L. Dignat-George F. (2006). Procoagulant microparticles: disrupting the vascular homeostasis equation? Arterioscler. Thromb. Vasc. Biol. 26 (12), 2594–2604. 10.1161/01.ATV.0000246775.14471.26 16990554

[B41] Ngambenjawong C. Gustafson H. H. Pun S. H. (2017). Progress in tumor-associated macrophage (TAM)-targeted therapeutics. Adv. Drug Deliv. Rev. 114, 206–221. 10.1016/j.addr.2017.04.010 28449873 PMC5581987

[B42] Noishiki C. Yuge S. Ando K. Wakayama Y. Mochizuki N. Ogawa R. (2019). Live imaging of angiogenesis during cutaneous wound healing in adult zebrafish. Angiogenesis 22 (2), 341–354. 10.1007/s10456-018-09660-y 30607697

[B43] Nussbaum S. R. Carter M. J. Fife C. E. DaVanzo J. Haught R. Nusgart M. (2018). An economic evaluation of the impact, cost, and medicare policy implications of chronic nonhealing wounds. Value Health. 21 (1), 27–32. 10.1016/j.jval.2017.07.007 29304937

[B44] Oberkersch R. E. Pontarin G. Astone M. Spizzotin M. Arslanbaeva L. Tosi G. (2022). Aspartate metabolism in endothelial cells activates the mTORC1 pathway to initiate translation during angiogenesis. Dev. Cell. 57 (10), 1241–1256. 10.1016/j.devcel.2022.04.018 35580611

[B45] Okuno Y. Nakamura-Ishizu A. Kishi K. Suda T. Kubota Y. (2011). Bone marrow-derived cells serve as proangiogenic macrophages but not endothelial cells in wound healing. Blood. 117 (19), 5264–5272. 10.1182/blood-2011-01-330720 21411758 PMC3357943

[B46] Pakshir P. Noskovicova N. Lodyga M. Son D. O. Schuster R. Goodwin A. (2020). The myofibroblast at a glance. J. Cell Sci. 133 (13), jcs227900. 10.1242/jcs.227900 32651236

[B47] Primo L. di Blasio L. Roca C. Droetto S. Piva R. Schaffhausen B. (2007). Essential role of PDK1 in regulating endothelial cell migration. J. Cell Biol. 176 (7), 1035–1047. 10.1083/jcb.200607053 17371830 PMC2064087

[B48] Quintero-Fabián S. Arreola R. Becerril-Villanueva E. Torres-Romero J. C. Arana-Argaez V. Lara-Riegos J. (2019). Role of matrix metalloproteinases in angiogenesis and cancer. Front. Oncol. 9, 1370. 10.3389/fonc.2019.01370 31921634 PMC6915110

[B49] Shabbir A. Cox A. Rodriguez-Menocal L. Salgado M. Van Badiavas E. (2015). Mesenchymal stem cell exosomes induce proliferation and migration of normal and chronic wound fibroblasts, and enhance angiogenesis *in vitro* . Stem Cells Dev. 24 (14), 1635–1647. 10.1089/scd.2014.0316 25867197 PMC4499790

[B50] Shan S. K. Lin X. Li F. Xu F. Zhong J. Y. Guo B. (2019). Exosomes and bone disease. Curr. Pharm. Des. 25 (42), 4536–4549. 10.2174/1381612825666191127114054 31775592

[B51] Shiratori H. Feinweber C. Luckhardt S. Linke B. Resch E. Geisslinger G. (2017). THP-1 and human peripheral blood mononuclear cell-derived macrophages differ in their capacity to polarize *in vitro* . Mol. Immunol. 88, 58–68. 10.1016/j.molimm.2017.05.027 28600970

[B52] Shook B. Xiao E. Kumamoto Y. Iwasaki A. Horsley V. (2016). CD301b+ macrophages are essential for effective skin wound healing. J. Invest. Dermatol. 136 (9), 1885–1891. 10.1016/j.jid.2016.05.107 27287183 PMC5727894

[B53] Sindrilaru A. Scharffetter-Kochanek K. (2013). Disclosure of the culprits: Macrophages-versatile regulators of wound healing. Adv. Wound Care. 2 (7), 357–368. 10.1089/wound.2012.0407 PMC3842885 24587973

[B54] Somanath P. R. Chen J. Byzova T. V. (2008). Akt1 is necessary for the vascular maturation and angiogenesis during cutaneous wound healing. Angiogenesis. 11 (3), 277–288. 10.1007/s10456-008-9111-7 18415691 PMC2677211

[B55] Song M. S. Salmena L. Pandolfi P. P. (2012). The functions and regulation of the PTEN tumour suppressor. Nat. Rev. Mol. Cell Biol. 13 (5), 283–296. 10.1038/nrm3330 22473468

[B56] Sorg H. Tilkorn D. J. Hager S. Hauser J. Mirastschijski U. (2017). Skin wound healing: An update on the current knowledge and concepts. Eur. Surg. Res. 58 (1-2), 81–94. 10.1159/000454919 27974711

[B57] Spinetti G. Sangalli E. Tagliabue E. Maselli D. Colpani O. Ferland-McCollough D. (2020). MicroRNA-21/PDCD4 proapoptotic signaling from circulating CD34(+) cells to vascular endothelial cells: A potential contributor to adverse cardiovascular outcomes in patients with critical limb ischemia. Diabetes care. 43 (7), 1520–1529. 10.2337/dc19-2227 32358022 PMC7305013

[B58] Sunderkötter C. Goebeler M. Schulze-Osthoff K. Bhardwaj R. Sorg C. (1991). Macrophage-derived angiogenesis factors. Pharmacol. Ther. 51 (2), 195–216. 10.1016/0163-7258(91)90077-y 1784630

[B59] Swindle M. M. Makin A. Herron A. J. Clubb F. J. Jr. Frazier K. S. (2012). Swine as models in biomedical research and toxicology testing. Vet. Pathol. 49 (2), 344–356. 10.1177/0300985811402846 21441112

[B60] Toita R. Shimizu E. Murata M. Kang J. H. (2021). Protective and healing effects of apoptotic mimic-induced M2-like macrophage polarization on pressure ulcers in young and middle-aged mice. J. Control. Release 330, 705–714. 10.1016/j.jconrel.2020.12.052 33388342

[B61] Ueta T. Ishihara K. Notomi S. Lee J. J. Maidana D. E. Efstathiou N. E. (2019). RIP1 kinase mediates angiogenesis by modulating macrophages in experimental neovascularization. Proc. Natl. Acad. Sci. U. S. A. 116 (47), 23705–23713. 10.1073/pnas.1908355116 31685620 PMC6876205

[B62] Unuvar Purcu D. Korkmaz A. Gunalp S. Helvaci D. G. Erdal Y. Dogan Y. (2022). Effect of stimulation time on the expression of human macrophage polarization markers. PloS one. 17 (3), e0265196. 10.1371/journal.pone.0265196 35286356 PMC8920204

[B63] van Hinsbergh V. W. Engelse M. A. Quax P. H. (2006). Pericellular proteases in angiogenesis and vasculogenesis. Arterioscler. Thromb. Vasc. Biol. 26 (4), 716–728. 10.1161/01.ATV.0000209518.58252.17 16469948

[B64] Veith A. P. Henderson K. Spencer A. Sligar A. D. Baker A. B. (2019). Therapeutic strategies for enhancing angiogenesis in wound healing. Adv. Drug Deliv. Rev. 146, 97–125. 10.1016/j.addr.2018.09.010 30267742 PMC6435442

[B65] Wilkinson H. N. Hardman M. J. (2020). Wound healing: cellular mechanisms and pathological outcomes. Open Biol. 10 (9), 200223. 10.1098/rsob.200223 32993416 PMC7536089

[B66] Yang H. Rudge D. G. Koos J. D. Vaidialingam B. Yang H. J. Pavletich N. P. (2013). mTOR kinase structure, mechanism and regulation. Nature 497 (7448), 217–223. 10.1038/nature12122 23636326 PMC4512754

[B67] Yang Y. Guo Z. Chen W. Wang X. Cao M. Han X. (2021). M2 macrophage-derived exosomes promote angiogenesis and growth of pancreatic ductal adenocarcinoma by targeting E2F2. Mol. Ther. 29 (3), 1226–1238. 10.1016/j.ymthe.2020.11.024 33221435 PMC7934635

[B68] Zhang J. Li S. Li L. Li M. Guo C. Yao J. (2015). Exosome and exosomal microRNA: trafficking, sorting, and function. Genomics Proteomics Bioinforma. 13 (1), 17–24. 10.1016/j.gpb.2015.02.001 PMC4411500 25724326

[B69] Zhang W. Bai X. Zhao B. Li Y. Zhang Y. Li Z. (2018). Cell-free therapy based on adipose tissue stem cell-derived exosomes promotes wound healing via the PI3K/Akt signaling pathway. Exp. Cell Res. 370 (2), 333–342. 10.1016/j.yexcr.2018.06.035 29964051

[B70] Zhang J. Muri J. Fitzgerald G. Gorski T. Gianni-Barrera R. Masschelein E. (2020). Endothelial lactate controls muscle regeneration from ischemia by inducing M2-like macrophage polarization. Cell Metab. 31 (6), 1136–1153. 10.1016/j.cmet.2020.05.004 32492393 PMC7267778

[B71] Zhang Y. Pan Y. Liu Y. Li X. Tang L. Duan M. (2021). Exosomes derived from human umbilical cord blood mesenchymal stem cells stimulate regenerative wound healing via transforming growth factor-β receptor inhibition. Stem Cell Res. Ther. 12 (1), 434. 10.1186/s13287-021-02517-0 34344478 PMC8336384

[B72] Zhang Y. Yan J. Liu Y. Chen Z. Li X. Tang L. (2021). Human amniotic fluid stem cell-derived exosomes as a novel cell-free therapy for cutaneous regeneration. Front. Cell Dev. Biol. 9, 685873. 10.3389/fcell.2021.685873 34235150 PMC8255501

[B73] Zheng P. Chen L. Yuan X. Luo Q. Liu Y. Xie G. (2017). Exosomal transfer of tumor-associated macrophage-derived miR-21 confers cisplatin resistance in gastric cancer cells. J. Exp. Clin. Cancer Res. 36 (1), 53. 10.1186/s13046-017-0528-y 28407783 PMC5390430

