# Supplementary material for: Exosomes derived from M2 macrophages induce angiogenesis to promote wound healing
Source: Front Mol Biosci. 2022 Oct 11;9:1008802. doi: 10.3389/fmolb.2022.1008802 (PMC9592913; doi:10.3389/fmolb.2022.1008802)
Supplement: Supplementary file 1 [file DataSheet1.DOCX]

**Supporting Figure 1: Statistics of tube formation assay in Figure 3H**


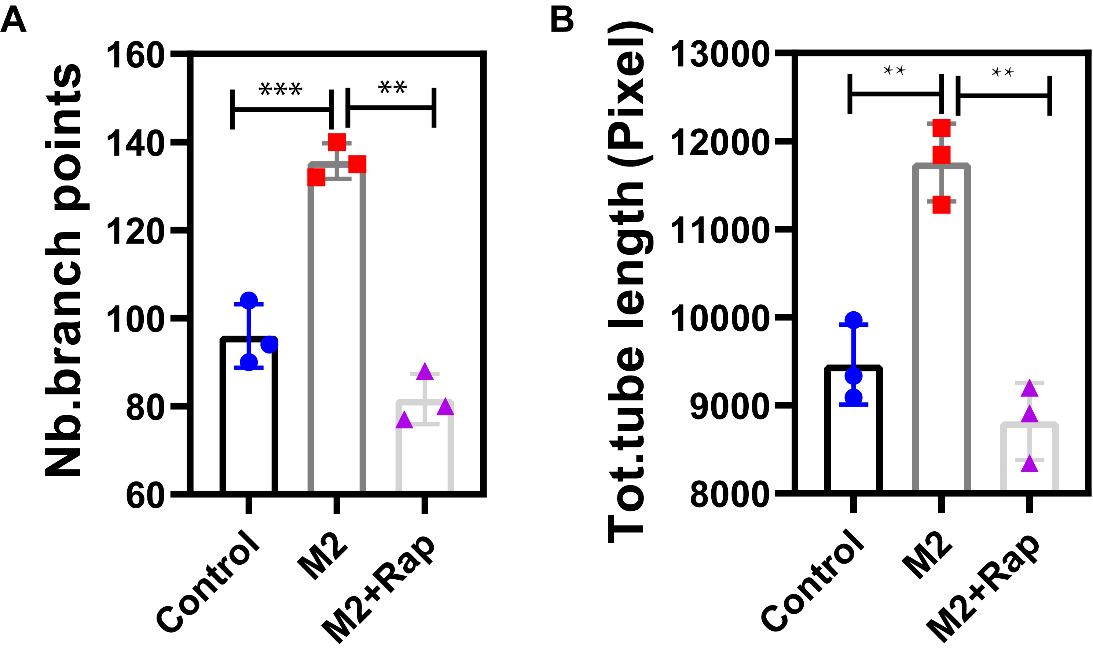


**Figure S1. A) & B) Statistics of tube formation assay presented in Figure 3H.** After 16h standard culturing, the number of branch points and the length of the total tube were analyzed by ImageJ. Rap + M2-EXO co-treated group had extremely shorter lengths of total tube and fewer branch points, which indicated that Rap counteracted the angiogenesis role induced by M2-EXO by inhibiting the mTOR pathway. Data was expressed as mean ± SD (n = 3) *= significant, * p < 0.05; **, p < 0.01,*** p <0.005; p ****<0.001, Student t-test)

**Supporting Figure 2:** After testing miR-21 expression in exosomes derived from normal M2 and miR-21 inhibitor transfected M2, we found that miR-21 inhibitor reduces miR-21 level in M2-EXO inh.


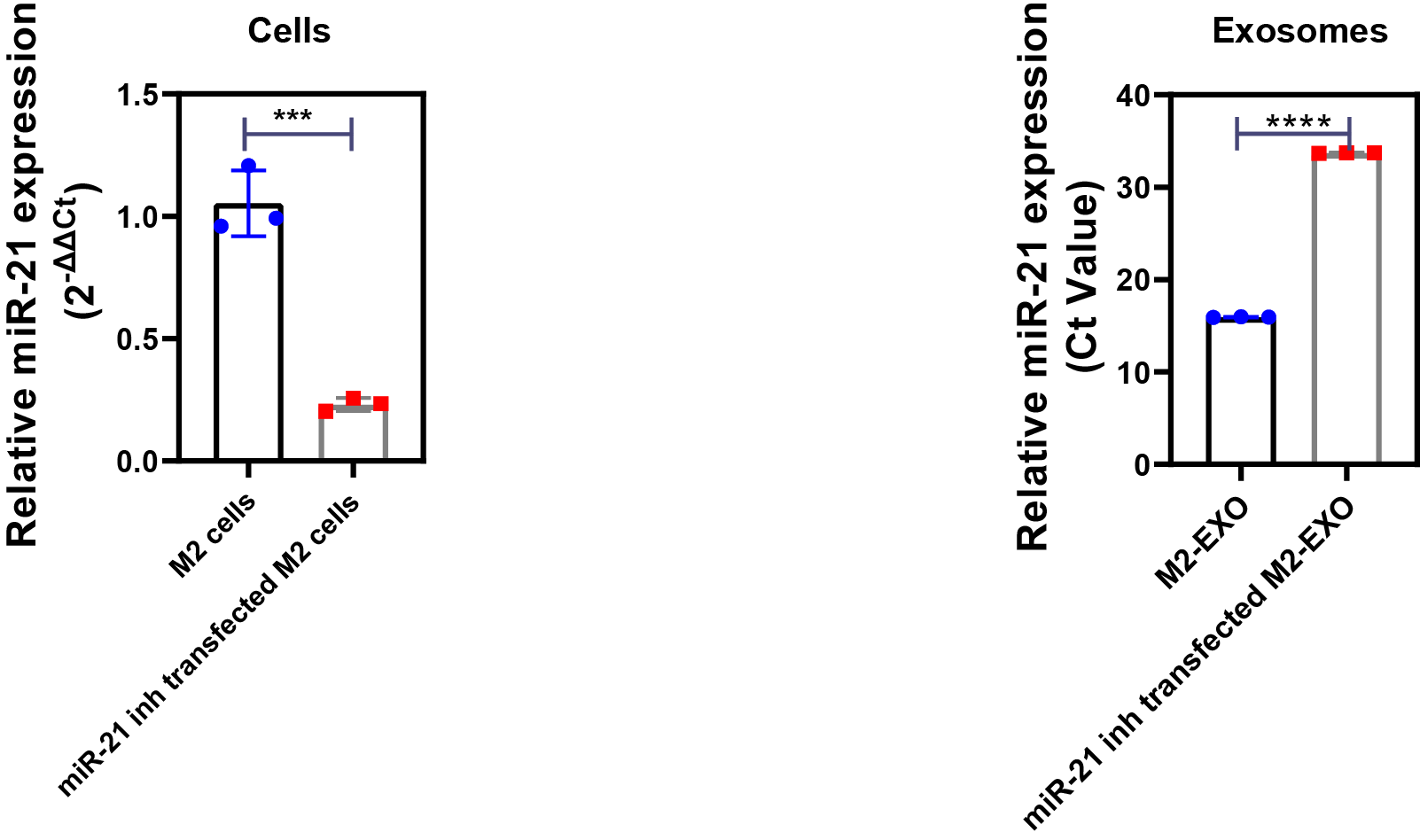


**Figure S2. Left**) qRT-PCR was used to test miR-21 expression level in Type 2 macrophages and miR-21 inhibitor transfected Type 2 macrophages. Results were shown by 2^-ΔΔCt^, and U6 was employed as a reference gene; **Right)** qRT-PCR was used to test miR-21 expression levels in exosomes, respectively derived from Type 2 macrophages and miR-21 inhibitor transfected Type 2 macrophages. Without proper reference gene, results were presented by Ct value. With a higher Ct Value, exosomes derived from miR-21 inhibited M2 cells had a lower miR-21 expression level, which proved that the transfection of miR-21 inhibitor significantly reduced miR-21 level in exosomes. Data was expressed as mean ± SD (n = 3) *= significant, *** p <0.005; p ****<0.001, Student t-test)

**Supporting Figure 3:** Statistical analysis of tube formation assay in Figure 5G


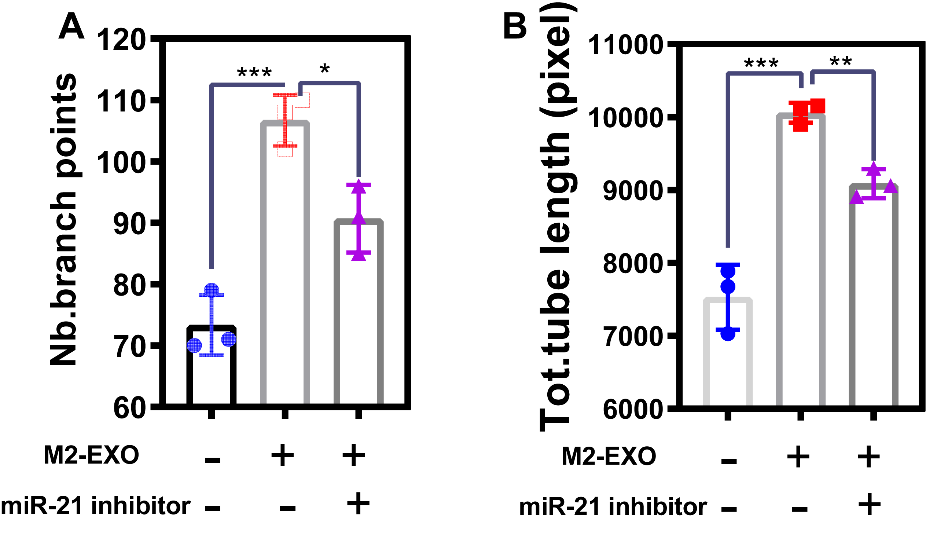


**Figure S3**. **A)&B)** Statistics of tube formation assay were presented in **Figure 5G**. The number of branch points was employed as a co-indicator with lengths of the total tube to evaluate the angiogenesis ability. miR-21 inhibitor + M2-EXO co-treated group had fewer branch points, which indicated that M2-EXO acted on angiogenesis by increasing miR-21 in HUVECs. Data was expressed as mean ± SD (n = 3) *= significant, * p < 0.05; *** p <0.005, Student t-test)

**Supporting Figure 4: Bio-safety test**


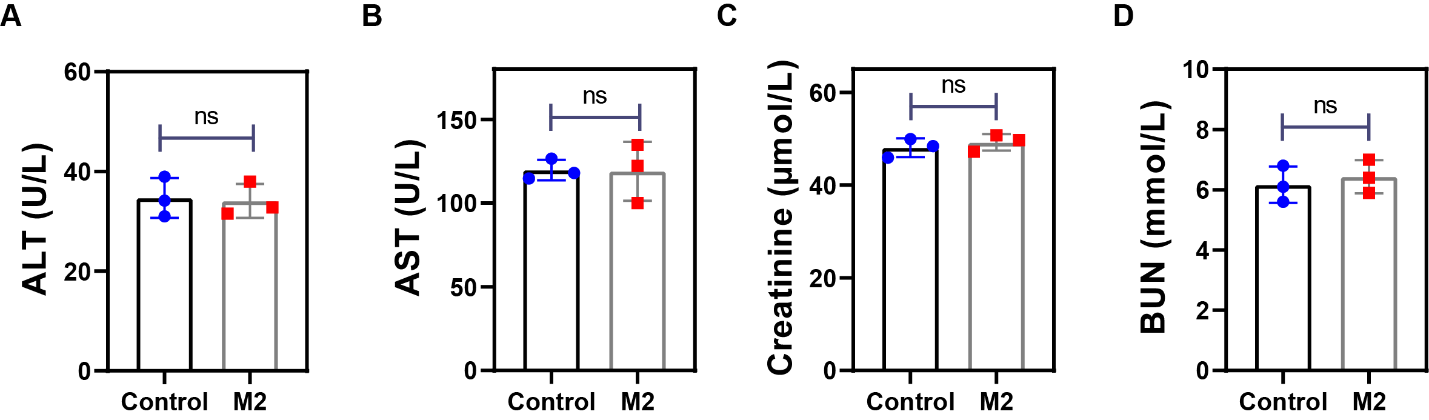


**Figure S4. A)-D)** The liver (ALT and AST) and renal (Creatinine and BUN) function indicators were used to evaluate the biosafety of M2-EXO. ELISA was employed to test these indicators in mice serum. Results showed that there are no statistical differences between the serological indicators of the control group and the M2-EXO treated group. Data were expressed as mean ± SD (n = 3) ns=no statistical differences, Student t-test)
